# DBC1, p300, HDAC3, and Siah1 coordinately regulate ELL stability and function for expression of its target genes

**DOI:** 10.1073/pnas.1912375117

**Published:** 2020-03-09

**Authors:** Subham Basu, Mahesh Barad, Dipika Yadav, Arijit Nandy, Bidisha Mukherjee, Jit Sarkar, Partha Chakrabarti, Satinath Mukhopadhyay, Debabrata Biswas

**Affiliations:** ^a^Laboratory of Transcription Biology, Molecular Genetics Division, Council of Scientific and Industrial Research (CSIR)–Indian Institute of Chemical Biology (IICB), Kolkata 700032, India;; ^b^Department of Endocrinology and Metabolism, Institute of Postgraduate Medical Education & Research (IPGM&R), Kolkata 700020, India;; ^c^Metabolic Diseases Laboratory, Cell Biology and Physiology Division, CSIR–IICB, Kolkata 700032, India

**Keywords:** transcription, ELL, acetylation, ubiquitylation, DBC1

## Abstract

Among all of the Super Elongation Complex (SEC) components, ELL1 (also known as ELL) is the only bona fide elongation factor that directly stimulates transcription elongation by RNA polymerase II. However, the mechanism(s) of functional regulation of ELL1 (referred to as ELL hereafter), through its stabilization, is completely unknown. Here, we report a function of human DBC1 in regulating ELL stability involving HDAC3, p300, and Siah1. Mechanistically, we show that p300-mediated site-specific acetylation increases, whereas HDAC3-mediated deacetylation decreases, ELL stability through polyubiquitylation by the E3 ubiquitin ligase Siah1. DBC1 competes with HDAC3 for the same binding sites on ELL and thus increases its acetylation and stability. Knockdown of DBC1 reduces ELL levels and expression of a significant number of genes, including those involved in glucose metabolism. Consistently, Type 2 diabetes patient-derived peripheral blood mononuclear cells show reduced expression of DBC1 and ELL and associated key target genes required for glucose homeostasis. Thus, we describe a pathway of regulating stability and functions of key elongation factor ELL for expression of diverse sets of genes, including ones that are linked to Type 2 diabetes pathogenesis.

## Significance

Human RNA polymerase II (Pol II) transcribes messenger RNA from underlying DNA sequences for the purpose of generating proteins for proper biological functions. One of the key steps of Pol II-driven transcription is elongation, which is directly stimulated by transcription factors such as ELL and affects expression of vast number of genes. However, mechanisms by which the overall functions of ELL are regulated through its stabilization are completely unknown. In this study, we decipher a mechanism of regulation of ELL stability, and thus its functions through multiple factors involving DBC1, HDAC3, p300, and Siah1 for regulation of expression of diverse sets of genes that are required for several physiological processes, including maintenance of glucose homeostasis in human.

During the last few decades, studies in the field of transcriptional regulation were focused on detailed understanding of factors involved, and their role, in regulation of the initiation step. Although transcriptional initiation plays a rate-limiting step for controlling expression of a significant number of genes, studies in the last several years have also shown a role for postinitiation steps, especially at the step of promoter proximal pausing, in overall regulation of transcription of genes that are important for development (1).

Among the elongation factors/complexes, human Super Elongation Complex (SEC) has been proposed to have multiple elongation factors in a megadalton complex (2, 3). Predominant players of SEC are AF9, ENL, ELL/ELL2, AFF1, AFF4, and P-TEFb complex (a heterodimer of CyclinT1 and CDK9). Excepting P-TEFb complex, other SEC components are frequently fused with the N terminus of Mixed Lineage Leukemia 1 (MLL1, hereafter MLL) to give rise to MLL fusion proteins and cause leukemogenesis (4).

Among all of the SEC components, ELL is the only bona fide elongation factor that directly stimulates transcription elongation by RNA polymerase II (hereafter Pol II) in vitro by reducing the rate of Pol II stalling during elongation (5–7). ELL interacts with ELL-associated factors (EAF1/2) that directly stimulate ELL-mediated transcription elongation by Pol II (2, 7–10). Besides SEC, ELL also associates with EAF1, KIAA0947 (aka ICE1), and NARG2 (aka ICE2) to form Little Elongation Complex (LEC) that functions in regulating small nuclear RNA gene transcription by Pol II as well as in the recruitment of TFIIH during transcription-coupled DNA repair (11–13). Although mechanisms of ELL2 degradation by the E3 ubiquitin ligase, Siah1, have been described by two earlier studies (14, 15), regulation of ELL function through its stabilization is completely unknown. In this regard, we were intrigued by the strong association of Deleted in Breast Cancer 1 (DBC1) in our ELL.com mass spectrometric analysis (2) and *SI Appendix*, Fig. S1*A*.

Human DBC1 was first described as a negative regulator of deacetylase activity of the histone deacetylase (HDAC), SIRT1, thus protecting the acetylation of tumor suppressor p53, a key modification required for its function within mammalian cells (16, 17). DBC1 has also been shown to regulate p53 stability through its competitive binding with E3 ubiquitin ligase MDM2 (18). Physiologically, DBC1 knockout mice show high fat diet-induced liver steatosis through regulation of SIRT1 activity as well as an increase in blood glucose level through increased gluconeogenesis (19, 20). A recent study has also shown a role for the Nudix homology domain of DBC1 in regulating PARP binding in a NAD+ concentration-dependent manner (15, 21). However, beyond its role in regulating p53 activity by controlling SIRT1 deacetylase functions, the role of DBC1 in regulating other key transcription factors have not been documented in detail.

In this study, we describe a role of DBC1 in regulation of ELL stability, and thus its function(s) within mammalian cells that also involves the activity of critical regulators such as p300, HDAC3, and Siah1. Our detailed studies have uncovered a pathway of transcriptional regulation involving the p300/DBC1/HDAC3/Siah1/ELL axis within mammalian cells that may have an implication for proper expression of key genes required for several physiological processes, including glucose homeostasis, in human.

## Results

### Interaction between ELL and DBC1.

**Fig. 1.**
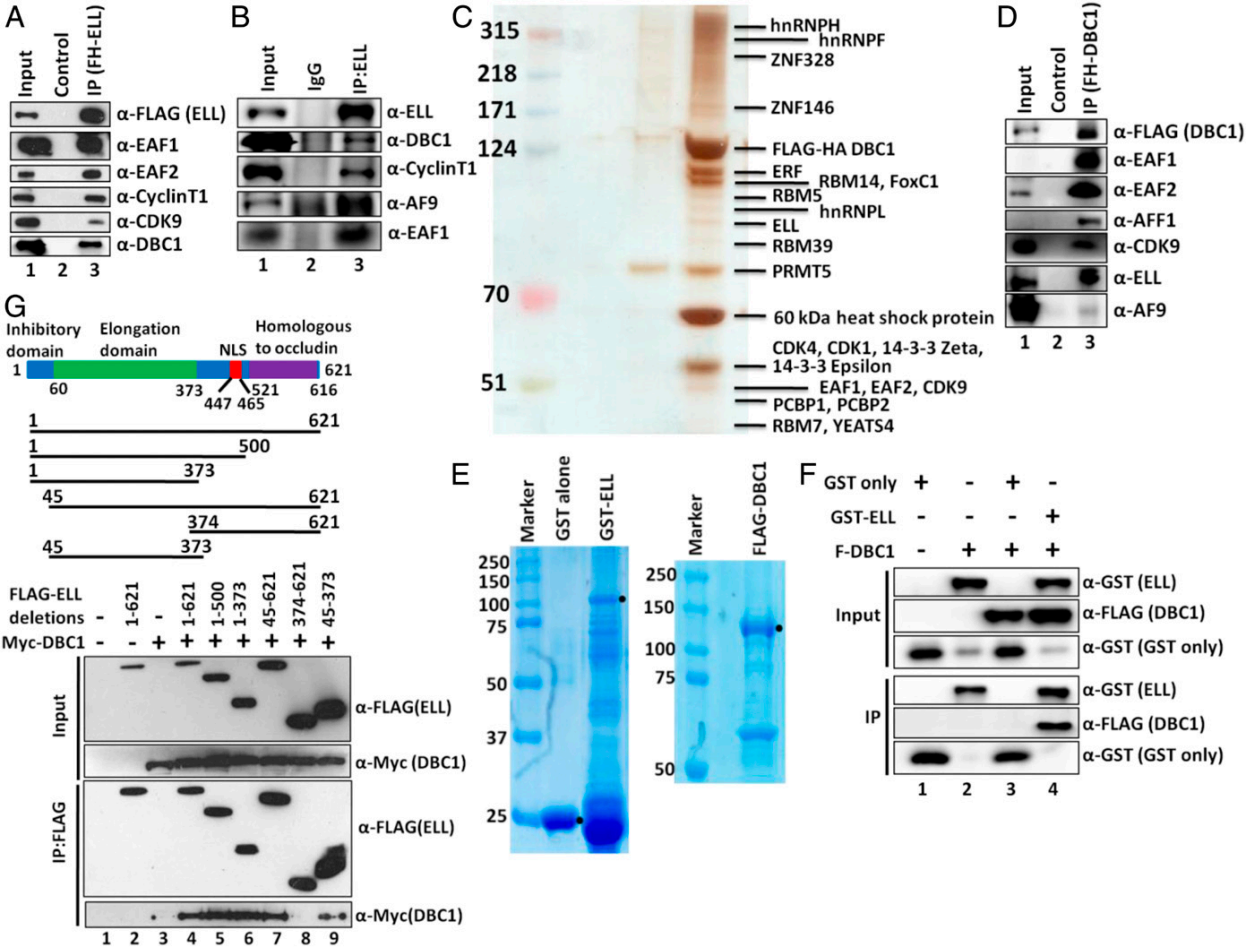
DBC1 and ELL interact both in vitro and in vivo within mammalian cells. (*A* and *B*) IP and Western blotting analysis showing identification of DBC1 as an interactor of (*A*) ectopic and (*B*) endogenous ELL along with other known interacting components (IgG: immunoglobulin G). (*C*) Purification of DBC1-associated protein complex from a stable cell line that ectopically expresses DBC1 as FLAG-HA−tagged. Eluted proteins were run on 4 to 12% gradient gel and silver-stained for their visualization. Individual bands were excised, and proteins were identified by mass spectrometric analysis. (*D*) IP and Western blotting analysis for identification of SEC component association with DBC1. (*E*) Coomassie staining of purified recombinant GST, GST–ELL (using bacterial expression system), and FLAG-DBC1 (using baculoviral expression system). GST–ELL shows significant degradation in our purification. (*F*) In vitro direct interaction assay with purified proteins showing direct interaction of DBC1 with GST–ELL but not GST alone. (*G*) Cartoon diagram showing domains of ELL that are important for regulating its functions (*Upper*) (NLS: nuclear localization signal). Depiction of ELL domains that have been used for its interaction assay with DBC1 by co-IP analysis (*Middle*). Western blot analysis showing interaction of indicated domains of ELL with DBC1 within mammalian cells by co-IP analysis (*Lower*).

We reported association of SEC components with ELL in our earlier study (2). Along with SEC components, we also observed association of DBC1 with ELL in our mass spectrometric analysis (*SI Appendix*, Fig. S1*A*). Subsequent immunoprecipitation (IP) and blotting analysis using nuclear extract from a stable cell line expressing FLAG-HA–ELL protein confirmed ELL interaction with DBC1 as well as other known ELL interactors (Fig. 1*A*). IP of endogenous ELL using ELL-specific antibody and subsequent blotting analyses further confirmed ELL interaction with DBC1 in an endogenous context (Fig. 1*B*). Reciprocal IP using nuclear extract from a FLAG-HA−DBC1 expressing stable cell line (*SI Appendix*, Fig. S1*B*) and subsequent mass spectrometric and immunoblotting analyses further confirmed DBC1 interaction with ELL (Fig. 1 *C* and *D*). Interestingly, besides ELL, both of the analyses also showed DBC1 interaction with other SEC components (Fig. 1 *C* and *D*). Subsequently, using purified recombinant DBC1 and ELL proteins through their expression in baculoviral and bacterial expression system, respectively (Fig. 1*E*), in vitro interaction analysis further confirmed direct interaction of DBC1 with GST–ELL, but not GST alone (Fig. 1*F*, lane 3 vs. lane 4). Therefore, based on these analyses, we conclude that human DBC1 and ELL interact directly with each other both in vitro as well as in vivo within mammalian cells.

Next, we were interested in identifying ELL domain(s) that would interact with DBC1. Coimmunoprecipitation (co-IP) analyses using ELL deletion constructs cloned in a mammalian expression vector (Fig. 1 *G*, *Middle*) showed that fragments containing the elongation domain (45 to 373) interacted with DBC1 (Fig. 1*G*, IP panel, lanes 4 to 7 and lane 9), whereas elongation-deficient domain (374 to 621) failed to do so (Fig. 1*G*, lane 8). Therefore, we conclude that the elongation domain of ELL is involved in its interaction with DBC1 within mammalian cells.

### DBC1 and ELL Positively Regulate Transcription of Chromosomally Integrated Reporter Gene Expression.

Toward understanding the functional significance of the role of DBC1 and ELL in regulation of Pol II-mediated transcription, we initially sought to use a chromosomally integrated reporter gene expression system. This system contains the adenovirus major late promoter followed by five copies of GAL4 DNA binding sites upstream of a reporter luciferase gene (22, 23). Since the reporter gene is expressed in context of chromatin, as opposed to a naked DNA template, the expression analysis reflects function of any given protein/complex similar to their native target genes in vivo. Our initial analysis showed that, with increasing expression, DBC1 strongly stimulated target reporter gene activation in the presence of activator GAL4–VP16 (*SI Appendix*, Fig. S2*A*). This effect is direct, since DBC1 failed to show any effect on the expression of GAL4–VP16 (*SI Appendix*, Fig. S2*B*). Further, consistent with its role in positive regulation of transcription, we observed a strong effect of ELL in stimulating reporter gene expression in the presence of GAL4–VP16 (*SI Appendix*, Fig. S2*C*). Therefore, we conclude that both the human DBC1 and ELL have positive roles in regulation of target chromosomally integrated reporter gene expression.

Our subsequent analysis through coexpression of increasing DBC1 and constant ELL further showed increased reporter gene activity compared with ELL alone (*SI Appendix*, Fig. S2*D*). The observed effect is additive rather than synergistic. Similar results with increasing ELL expression in the presence of constant DBC1 (*SI Appendix*, Fig. S2*E*) led us to conclude that both DBC1 and ELL positively regulate transcription of chromosomally integrated reporter gene in an additive manner.

### DBC1 Stabilizes ELL Protein within Mammalian Cells.

**Fig. 2.**
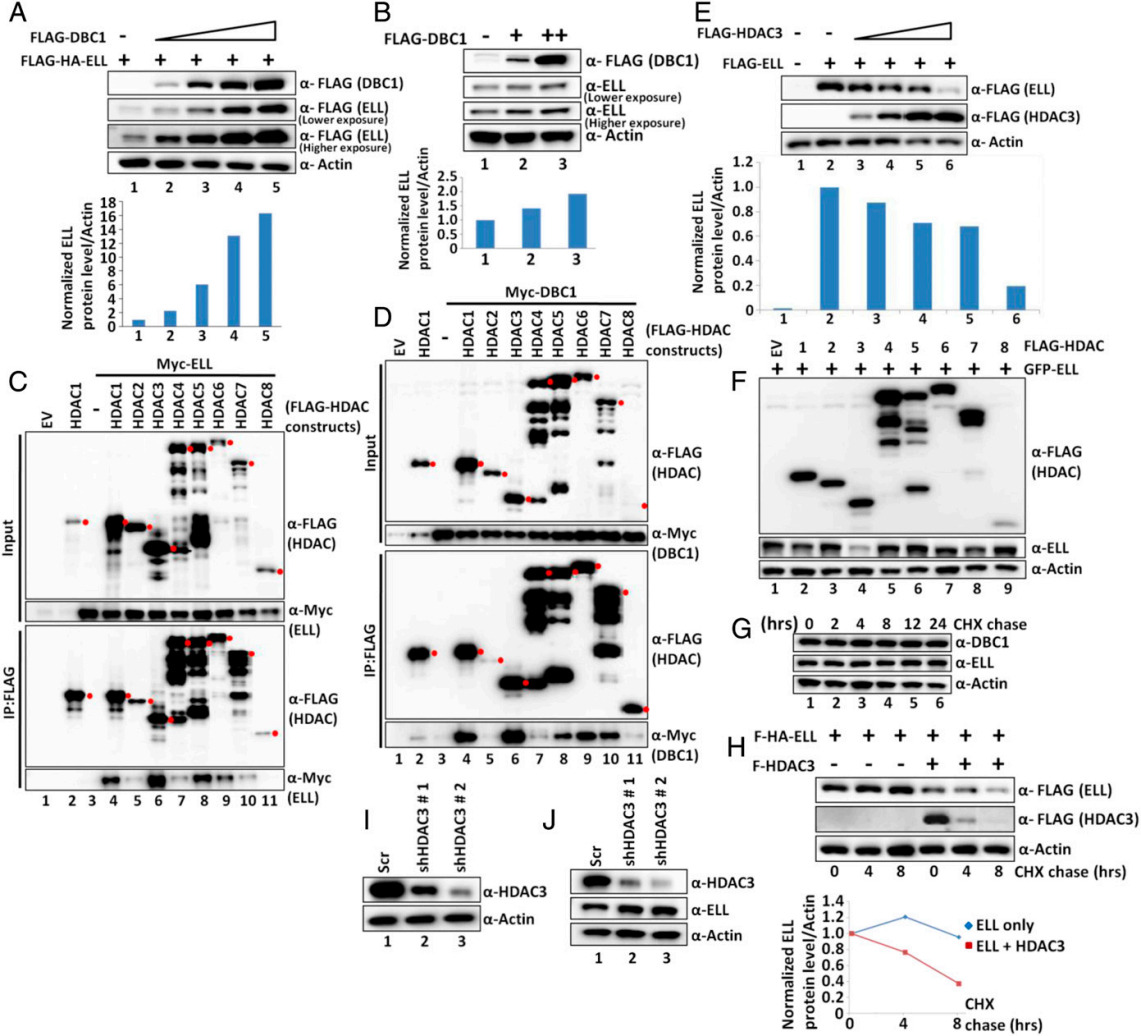
DBC1 and HDAC3 act in opposition to regulate ELL stability within mammalian cells. (*A* and *B*) Western blot analysis showing the effect of overexpression of DBC1 on (*A*) ectopic and (*B*) endogenous expression of ELL in 293T cells (*Upper*) and quantitation of ELL protein relative to Actin (*Lower*). (*C* and *D*) Co-IP and Western blot analysis showing interactions of HDAC1-8 with (*C*) ELL and (*D*) DBC1 in 293T cells. Because of the unavailability of human HDAC2, murine HDAC2 was used in this assay. Red filled circles indicate target protein bands, and others are either degradation or nonspecific proteins. (*E*) Western blot analysis showing the effect of overexpression of HDAC3 on ectopic expression of ELL in 293T cells (*Upper*) and quantitation of ELL relative to Actin (*Lower*). (*F*) Western blot analysis showing effect of overexpression of HDAC1-8 on ectopic expression of ELL in 293T cells. (*G*) CHX chase assay showing degradation kinetics of endogenous DBC1 and ELL at different time points after addition of CHX in 293T cells. (*H*) CHX chase assay showing degradation kinetics of ectopically expressed ELL in presence or absence of ectopically expressed HDAC3 at different time points after addition of CHX in 293T cells (*Upper*) and quantitation of ELL degradation over time (*Lower*). (*I* and *J*) Western blotting analysis showing (*I*) the stable knockdown of HDAC3 and (*J*) its effect on expression of endogenous ELL by using two different shRNAs in 293T cells.

The interesting and consistent observation of increased expression of ELL protein upon increasing coexpression of DBC1 (*SI Appendix*, Fig. S2*D*, compare lane 4 with lanes 5 to 8), despite using a constant amount of ELL expression construct in our reporter assay, further prompted us to investigate whether DBC1 could have any role(s) in stabilizing ELL protein. As shown in Fig. 2*A*, increased expression of DBC1 resulted in a marked increase in expression of cotransfected ELL protein (Fig. 2*A* and *Lower* quantitation), but not control GFP (*SI Appendix*, Fig. S3*A*). Although, through qRT-PCR analysis, increased DBC1 messenger RNA (mRNA) was observed upon increased DBC1 cotransfection (*SI Appendix*, Fig. S3 *B*, *Upper*), our failure to observe any significant effect on ELL mRNA expression (*SI Appendix*, Fig. S3 *B*, *Lower*) indicated an overall DBC1 effect on ELL protein level only and ruled out any effect through increased ELL transcription. Our subsequent analysis further showed an effect of increased DBC1 expression on increasing endogenous ELL protein levels in both 293T and HCT116 cells (Fig. 2*B* and *SI Appendix*, Fig. S3*C*, respectively), thus ruling out a cell type-specific effect of DBC1 on ELL stability. Therefore, we conclude that human DBC1 stabilizes ELL protein within mammalian cells in a cell type-independent manner.

### HDAC3 Interacts with ELL and Causes Its Destabilization.

For a deeper mechanistic understanding of ELL stabilization, and since DBC1 regulates p53 functions through its association with SIRT1 (16–18), we initially tested whether ELL would also show an interaction with SIRT1. As shown in *SI Appendix*, Fig. S3*D*, in our co-IP analysis, we have failed to observe any interaction between ELL and SIRT1. However, subsequent co-IP analyses of ELL with other HDACs showed specific and strong interactions of ELL with HDAC1, HDAC3, and HDAC5 (Fig. 2*C*, lanes 4, 6, and 8), in which HDAC3 showed stronger interaction than the others. Interestingly, and consistent with an earlier study (24), similar analyses with DBC1 also showed specific and strong interaction with HDAC1 and HDAC3 (Fig. 2*D*, lanes 4 and 6). Therefore, we conclude that both ELL and DBC1 interact with HDAC3 more strongly than with other HDACs within mammalian cells, and subsequent studies were designed toward deeper understanding of the role of HDAC3 in regulation of DBC1-mediated ELL functions within mammalian cells.

Contrary to the effect of DBC1 on ELL, increased expression of HDAC3 resulted in decreased expression of ectopic ELL (Fig. 2*E*) without affecting its mRNA expression from the transfected plasmid as measured by qRT-PCR (*SI Appendix*, Fig. S3*E*). In fact, increased expression of HDAC3 caused an increase in ELL mRNA expression in our assay (*SI Appendix*, Fig. S3*E*; compare lane 2 with lanes 3 to 6). Similar effect of HDAC3 on ELL protein level in HCT116 cells (*SI Appendix*, Fig. S3*F*) also ruled out a cell type-specific effect. The effect of HDAC3 on ELL destabilization is very specific, since we failed to observe any significant effect of other HDACs when coexpressed with ELL (Fig. 2*F*). Further analysis through coexpression of SIRT1-7 proteins along with ELL also failed to show any specific and significant effect on ELL stability (*SI Appendix*, Fig. S3*G*). Thus, we conclude that human HDAC3 specifically destabilizes ELL protein without affecting transcription of ELL mRNA within mammalian cells. Consistent with a role of HDAC3 in regulation of ELL functions, we also observed endogenous HDAC3 interaction with ectopic ELL (*SI Appendix*, Fig. S3*H*) as well as endogenous ELL interaction with ectopic HDAC3 (*SI Appendix*, Fig. S3*I*).

Next, we addressed whether overexpression of HDAC3 would enhance ELL degradation kinetics within mammalian cells. Consistent with an earlier study showing ELL (ELL1) as a stable protein (14), our initial cycloheximide (CHX) chase assay showed unchanged levels of ELL and DBC1 even after 24 h of chase (Fig. 2*G*). However, overexpression of HDAC3 caused rapid degradation of ELL (Fig. 2*H*, quantitation in *Lower*) and thus led us to conclude that the stable human ELL protein is degraded significantly faster in the presence of overexpressed HDAC3.

Stable knockdown of HDAC3 by two different specific short hairpin RNAs (shRNAs) (Fig. 2*I*) (25) caused an increase in endogenous ELL protein level (Fig. 2*J*; compare lane 1 with lanes 2 to 3) without altering its mRNA level (*SI Appendix*, Fig. S3*J*), and thus further ruled out any artifacts in our observation using overexpressed HDAC3. Thus, we conclude that HDAC3 regulates degradation of the ectopic as well as endogenous ELL within mammalian cells.

### DBC1 and HDAC3 Act in Opposition to Stabilize ELL Protein.

**Fig. 3.**
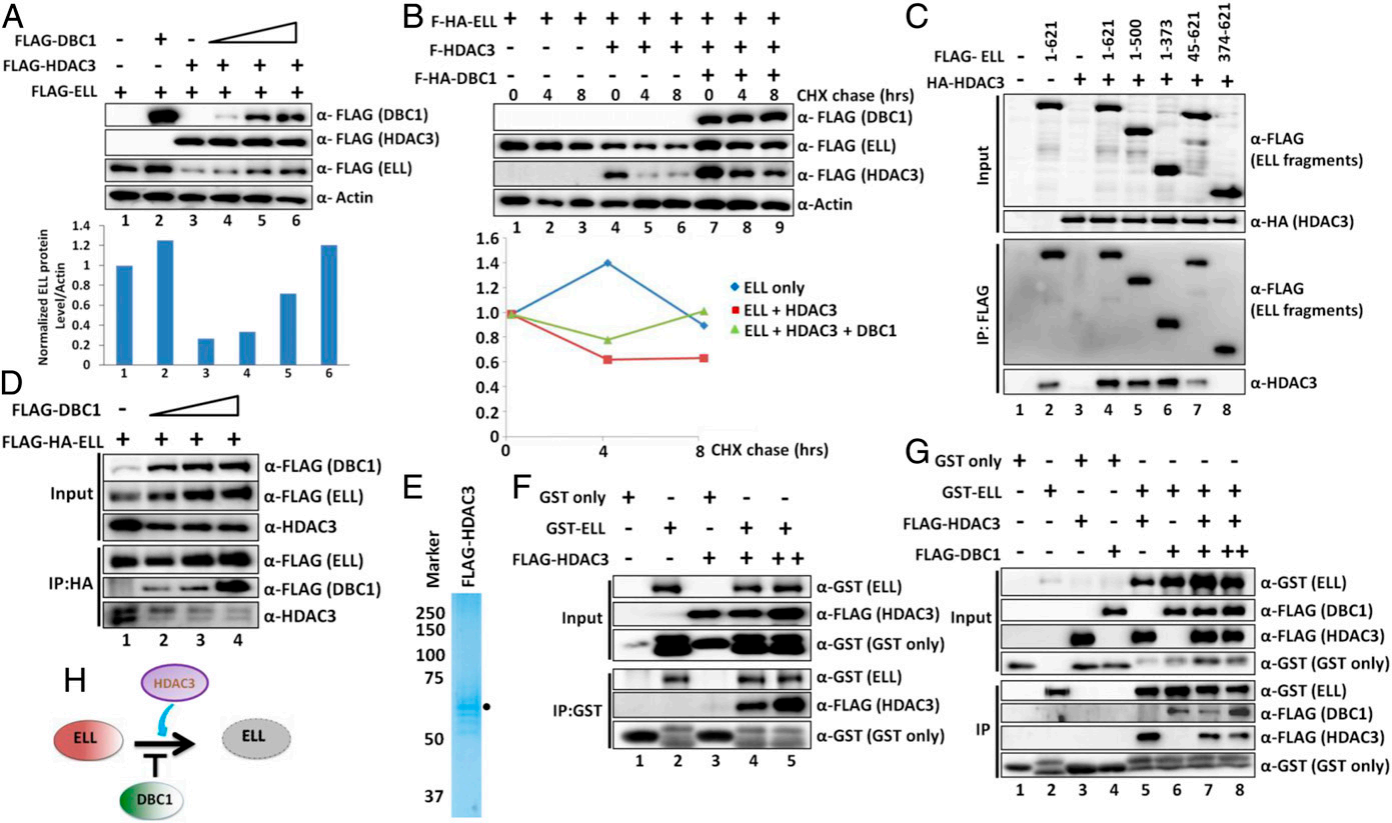
Competitive binding between DBC1 and HDAC3 to ELL. (*A*) Western blot analysis showing the rescue of HDAC3-mediated ELL degradation through concomitant overexpression of DBC1 (*Upper*) and quantitation of ELL protein relative to Actin (*Lower*). (*B*) CHX chase assay showing reduced degradation kinetics of ectopically expressed ELL mediated by HDAC3 in presence of DBC1 at different time points after addition of CHX in 293T cells (*Upper*) and quantitation of relative ELL degradation over time (*Lower*). (*C*) Western blot analysis showing the interaction of indicated ELL domains with HDAC3 within mammalian cells by co-IP analysis. (*D*) IP and Western blot analysis showing reduced interaction of HDAC3 with ELL upon increasing expression and binding of DBC1 to ELL within 293T cells. (*E*) Purification of HDAC3 through its overexpression in mammalian 293T cells. (*F*) In vitro direct interaction assay with purified proteins showing direct interaction of HDAC3 with GST–ELL but not GST alone. (*G*) In vitro direct binding assay, using purified proteins, showing competitive binding between HDAC3 and DBC1 to ELL. (*H*) Cartoon diagram depicting opposing roles of DBC1 and HDAC3 in regulation of ELL stability, wherein DBC1 stabilizes and HDAC3 destabilizes ELL protein.

So far, our analyses have shown opposing roles of DBC1 and HDAC3 toward ELL stabilization. Next, we addressed whether overexpression of DBC1 would rescue HDAC3-mediated ELL destabilization. As shown in Fig. 3*A*, although overexpression of HDAC3 destabilizes ELL (lane 3), concomitant expression of DBC1 rescued ELL protein from HDAC3-mediated degradation (compare lane 3 with lanes 4 to 6 and quantitation in *Lower*). Consistent with this opposing role, simultaneous coexpression of DBC1 significantly reduced ELL degradation kinetics in a CHX chase assay in the presence of ectopically expressed HDAC3 (Fig. 3*B*; compare lanes 4 to 6 with lanes 7 to 9 and quantitation in *Lower*). Therefore, based on all of these results, we conclude that human DBC1 and HDAC3 act in opposition, wherein DBC1 stabilizes and HDAC3 destabilizes ELL protein.

### DBC1 and HDAC3 Compete with Each Other for ELL Binding.

For further understanding the opposing roles of DBC1 and HDAC3 in regulating ELL stability, and primarily based on earlier observations showing DBC1 and MDM2 regulating p53 stability through their competitive binding (18), we wondered whether similar mechanisms would also exist in our study. To address this hypothesis, we initially identified ELL domain(s) that would interact with HDAC3. Co-IP analysis showed that, whereas an N-terminal fragment containing elongation domain fully retained HDAC3 interaction similar to that of full-length ELL (Fig. 3*C*, lane 6), a C-terminal fragment (374 to 621) failed to do so (Fig. 3*C*, lane 8). Thus, we conclude that human ELL interacts with HDAC3 through its N-terminal elongation domain. A similar interaction pattern of ELL and DBC1 (Fig. 1*G*) further indicated the existence of competition between these two factors for their binding to ELL. Consistently, whereas, in the absence of ectopically expressed DBC1, ELL interacted strongly with HDAC3 (Fig. 3*D*, lane 1), increasing expression and binding of ectopic DBC1 with ELL resulted in a concomitant reduction of HDAC3 binding (Fig. 3*D*; compare lane 1 with lanes 2 to 4). Toward obtaining direct evidence of similar competitive binding, we purified HDAC3 through its overexpression in mammalian cells and purification using a high salt-based purification method (Fig. 3*E*). Subsequent in vitro interaction showed that only purified recombinant GST–ELL interacted with HDAC3, not GST alone (Fig. 3*F*; compare lane 3 with lanes 4 to 5 in IP panel). Further assays using purified DBC1 (Fig. 1*E*) clearly showed that, whereas GST–ELL retained its strong interaction with HDAC3 (Fig. 3*G*, lane 5 in IP panel), addition of DBC1 and its binding strongly inhibited HDAC3 binding with GST–ELL (Fig. 3*G*; compare lane 5 with lanes 7 to 8 in IP panel). Therefore, all of these results led us to conclude that DBC1 and HDAC3 compete for the same binding site for their interaction with ELL and thus perform opposing functions in regulating ELL stability within mammalian cells (Fig. 3*H*).

### p300-Mediated Acetylation Increases ELL Stability.

**Fig. 4.**
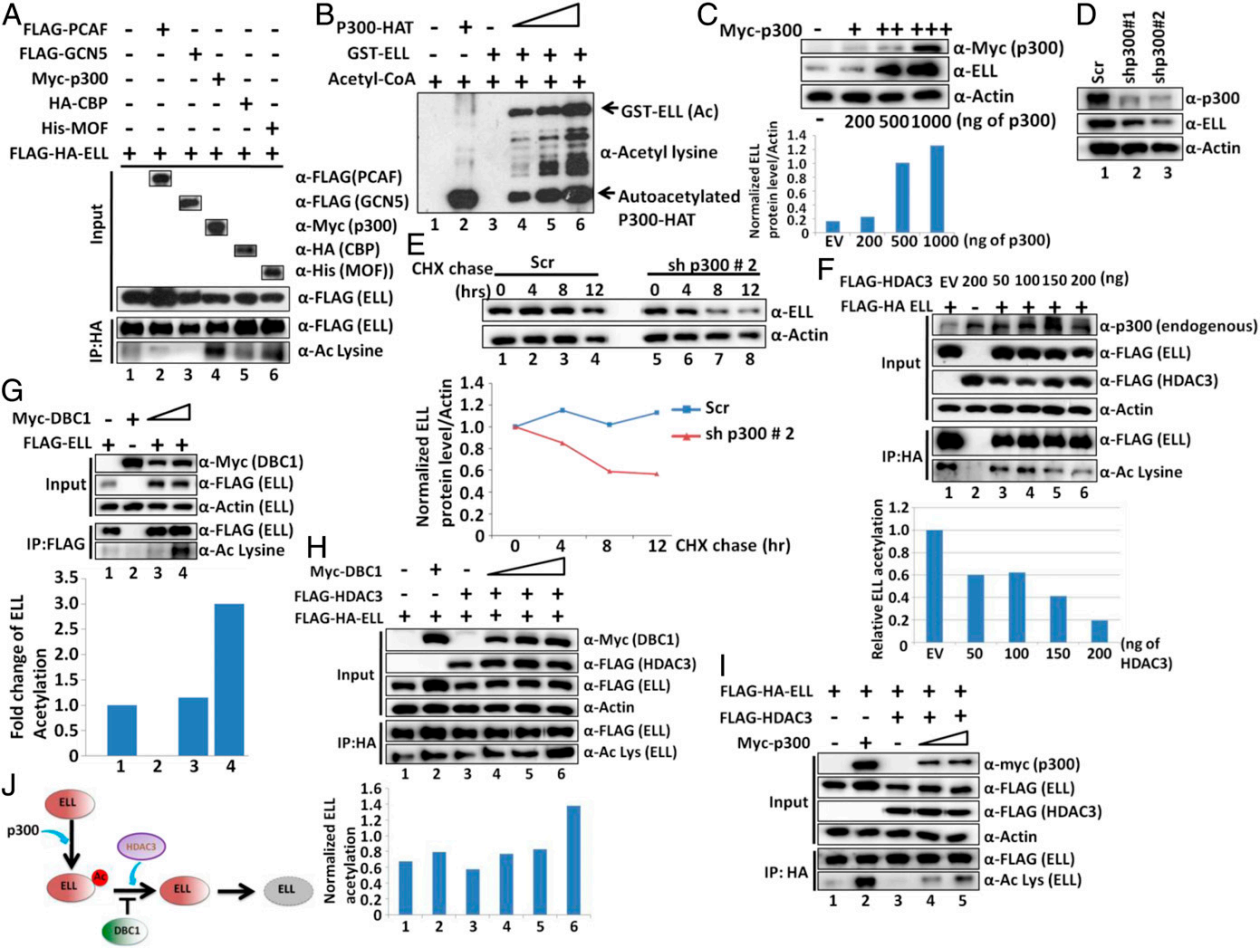
The p300-mediated acetylation stabilizes ELL, whereas DBC1 and HDAC3 act in an opposite manner to regulate ELL acetylation and stability. (*A*) Immunoblotting analyses showing robust ELL acetylation specifically by p300 within 293T cells through immunoblotting using pan-acetyl lysine-specific antibody. (*B*) In vitro HAT assay and subsequent Western blotting showing ELL acetylation by p300 using purified recombinant p300 HAT domain and GST–ELL (*SI Appendix*, Fig. S4*B*). (*C*) Western blot analysis showing the effect of overexpression of p300 on expression of endogenous ELL in 293T cells (*Upper*) and quantitation of ELL protein relative to Actin (*Lower*). (*D*) Western blot analysis showing the effect of stable p300 knockdown, by two different shRNA constructs, on endogenous ELL protein level within 293T cells. (*E*) CHX chase assay showing degradation kinetics of ELL in a p300 knockdown cell line when compared to control scramble cells (*Upper*). Quantitation of ELL protein degradation relative to Actin over time (*Lower*). (*F*) Western blot analysis showing deacetylation of ELL through overexpression of HDAC3 within 293T cells (*Upper*). Quantitation of relative ELL acetylation (*Lower*). (*G*) Western blot analysis showing increased ELL acetylation upon overexpression of DBC1 in 293T cells. (*H*) Western blot analysis showing rescue of acetylation of ELL, which is being deacetylated by HDAC3, through concomitant overexpression of DBC1 (*Upper*). Quantitation of relative ELL acetylation (*Lower*). (*I*) Western blot analysis showing rescue of acetylation of ELL, which is being deacetylated by HDAC3, through concomitant overexpression of p300. (*J*) Cartoon diagram depicting role of p300-mediated acetylation in regulation of ELL stability involving DBC1 and HDAC3.

Since HDAC3 destabilizes ELL at the protein level, we hypothesized that ELL protein may be destabilized by the deacetylation activity of HDAC3, whereas acetylation would increase its stability. Toward addressing this hypothesis, we initially tested whether ELL is acetylated within mammalian cells. Coexpression of ELL with various histone acetyl transferases and subsequent IP and blotting analysis using pan-acetyl lysine-specific antibody showed ELL acetylation by p300 and MOF (Fig. 4*A*, lanes 4 and 6) and not by PCAF or GCN5. Because of its maximum effect, we further focused on the role of p300-mediated ELL acetylation in regulating its functions. In our subsequent experiments, we observed increased ELL acetylation upon increased expression of p300 within mammalian cells, by cotransfection analysis (*SI Appendix*, Fig. S4*A*). For in vitro analysis of direct acetylation by p300, we purified recombinant p300 HAT domain through its expression in bacterial system (*SI Appendix*, Fig. S4*B*) (26). As shown in Fig. 4*B*, whereas purified recombinant ELL failed to show any acetylation signal by itself (lane 3), addition of p300 in the reaction showed strong ELL acetylation (compare lane 3 with lanes 4 to 6). Thus, we conclude that human p300 directly acetylates ELL in vitro as well as in vivo within mammalian cells.

Interestingly, contrary to the destabilization effect of HDAC3, concomitant expression of p300 increases ectopic ELL expression from a plasmid (*SI Appendix*, Fig. S4*C*, quantitation in *Lower*). A parallel analysis showed a robust effect of p300 overexpression on increasing endogenous ELL protein levels (Fig. 4*C*) without affecting its mRNA expression (*SI Appendix*, Fig. S4*D*). Based on these evidences, we conclude that p300-mediated acetylation increases ELL stability within mammalian cells.

Next, we knocked down endogenous p300 using two specific shRNAs (*SI Appendix*, Fig. S4*E*) (27) and observed a concomitant reduction in expression of endogenous ELL protein (Fig. 4*D*) without affecting its mRNA level (*SI Appendix*, Fig. S4*F*). A similar result is also observed for endogenous ELL protein levels upon p300 knockdown in HCT116 cells (*SI Appendix*, Fig. S4*G*), thus ruling out a cell type-specific effect. IP and subsequent blotting analysis also showed a reduced acetylation level of endogenous ELL and thus further confirmed a role for p300 in acetylating ELL (*SI Appendix*, Fig. S4*H*) in an endogenous context. Subsequent CHX chase analysis using these cells clearly showed faster degradation kinetics of endogenous ELL upon p300 knockdown than of control scramble cells (Fig. 4*E*). All of these analyses clearly confirm the role of p300 in stabilizing ELL within mammalian cells.

### HDAC3 Deacetylates ELL In Vitro and In Vivo.

To address opposing functions of p300-mediated acetylation and HDAC3-mediated deacetylation in regulation of ELL stability, we initially wondered whether ELL would be deacetylated by HDAC3. Coexpression and IP analysis showed that, with increased expression of HDAC3, the ELL acetylation level is concomitantly reduced (Fig. 4*F*, compare lane 1 with lanes 3 to 6). Since endogenous p300 levels remain unchanged upon increased expression of HDAC3 in our assay (Fig. 4 *F*, *Upper*, top row, input panel), an indirect effect of reduced p300 expression on overall decrease in ELL acetylation can be ruled out. Our subsequent in vitro deacetylation assay using purified and acetylated ELL showed modest decrease in ELL acetylation in the presence of HDAC3 (*SI Appendix*, Fig. S4*I*), and provided direct evidence of HDAC3-mediated ELL deacetylation. Further, pulling down endogenous ELL using ELL-specific antibody from control scramble and HDAC3 knockdown cells (Fig. 2*I*) and subsequent blotting analysis clearly showed an increase in endogenous ELL acetylation upon HDAC3 knockdown within mammalian cells (*SI Appendix*, Fig. S4*J*). Based on these results, we conclude that, whereas p300 acetylates ELL, acetylated ELL is subjected to deacetylation by HDAC3 in vitro as well as in vivo within mammalian cells.

### DBC1 and p300 Act in Opposition to HDAC3 to Regulate ELL Acetylation.

Since our results indicate an opposing function of DBC1 and HDAC3 in binding to ELL and regulating its stability, we wondered whether DBC1 would act against HDAC3 to increase ELL acetylation. Our initial experiments showed increased acetylation of ectopically expressed ELL when coexpressed with increasing DBC1 (Fig. 4*G*; compare lane 1 with lanes 3 to 4). Further, we addressed whether DBC1 could rescue HDAC3-mediated ELL deacetylation. As shown in Fig. 4*H*, whereas cotransfection of ELL with HDAC3 reduced its acetylation level (compare lane 1 with lane 3), concomitant expression of DBC1 rescued it (Fig. 4*H*; compare lane 3 with lanes 4 to 6). Similar results are also obtained upon overexpression of p300 within mammalian cells (Fig. 4*I*; compare lane 3 with lanes 4 to 5). Further, DBC1 overexpression markedly decreased endogenous ELL interaction with HDAC3 and increased its acetylation level (*SI Appendix*, Fig. S4*K*). Therefore, based on all of these results, we conclude that both DBC1 and p300 act against HDAC3 to regulate ELL acetylation, leading to its stability (Fig. 4*J*).

### HDAC3-Mediated ELL Destabilization Involves Ubiquitin Proteasome-Mediated Degradation System.

**Fig. 5.**
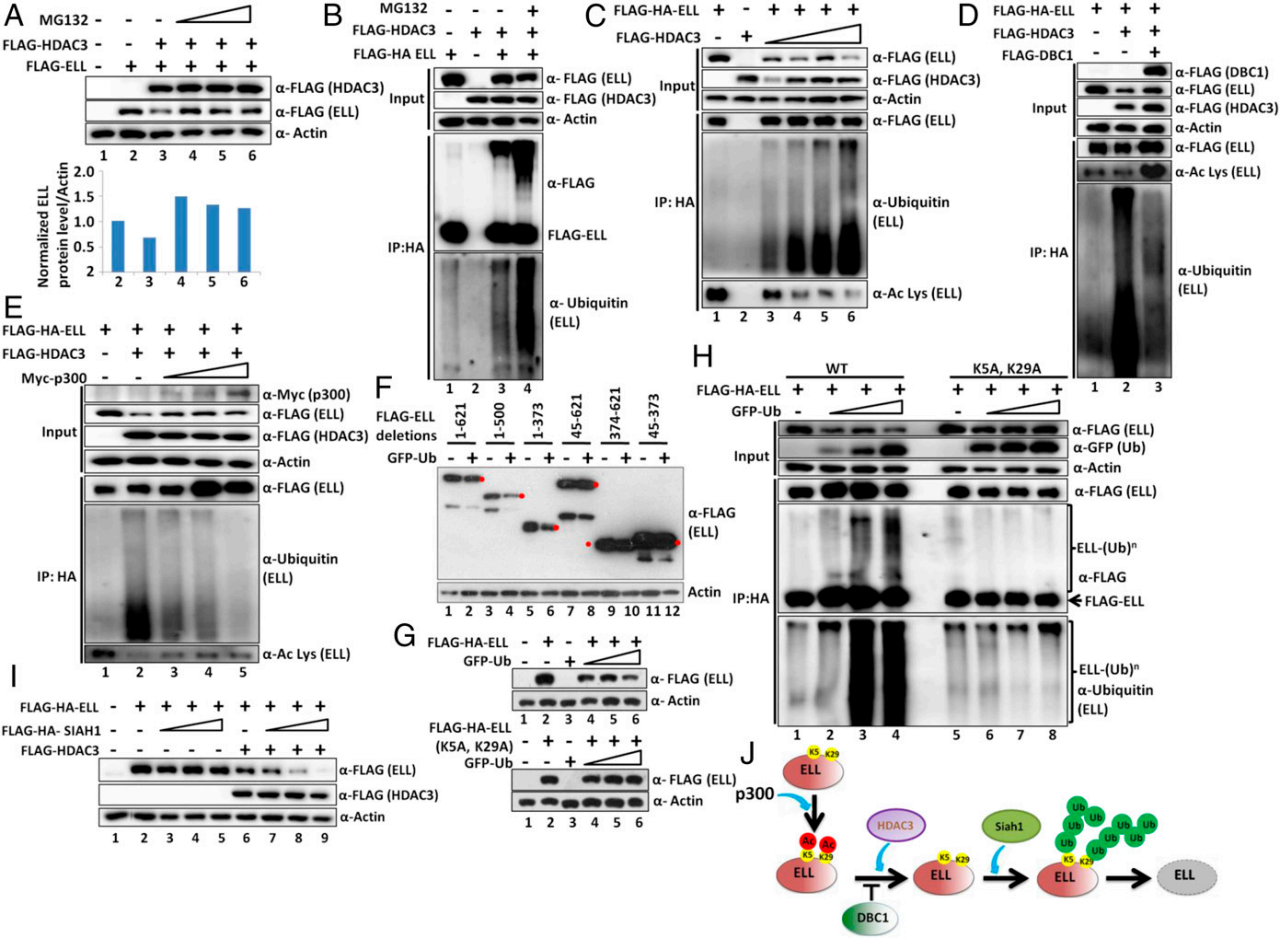
Poly-ubiquitylation of ELL at N-terminal K5 and K29 residues in presence of HDAC3 dictates ELL degradation by Siah1 within mammalian cells. (*A*) Western blot analysis showing rescue of HDAC3-mediated ELL degradation through addition of ubiquitin proteasome inhibitor MG132 (*Upper*) and quantitation of ELL level (*Lower*). (*B*) Western blot analysis showing increased polyubiquitylation of ELL by coexpression of HDAC3. The polyubiquitylation signal is further enhanced upon addition of MG132. (*C*) Western blot analysis showing a correlation of increased polyubiquitylation and decreased acetylation of ELL mediated by increased expression of HDAC3. (*D* and *E*) Western blot analysis showing strong correlation of increased ELL acetylation and reduced polyubiquitylation in presence of HDAC3 through concomitant overexpression of (*D*) DBC1 and (*E*) p300. (*F*) Western blot analysis showing the specific domain of ELL that shows sensitivity to ubiquitin-mediated degradation. Red filled circles indicate target protein bands, and others are either degradation or nonspecific proteins. (*G*) Western blot analysis showing sensitivity of ELL (WT) and ELL (K5A, K29A) mutant toward ubiquitin-mediated degradation. (*H*) Western blot analysis showing reduced polyubiquitylation of ELL (K5A, K29A) mutant in presence of overexpressed ubiquitin. (*I*) Western blot analysis showing increased ELL degradation by the ubiquitin E3 ligase Siah1 only in presence of HDAC3. (*J*) Cartoon diagram depicting the role of p300-mediated acetylation, DBC1-mediated acetylation protection, HDAC3-mediated deacetylation, and Siah1-mediated polyubiquitylation in overall regulation of ELL stability within mammalian cells.

For deeper understanding of the mechanisms involved in HDAC3-mediated ELL degradation, we initially used MG132 that inhibits ubiquitin proteasome-mediated degradation of target proteins and observed significant ELL stabilization from HDAC3-mediated degradation (Fig. 5*A*; compare lane 3 with lanes 4 to 6). This observation indicated a role for proteasome-mediated ubiquitylation in this degradation process. Consistent with this, whereas ectopically expressed ELL did not show much ubiquitylation in our assay (Fig. 5*B*, lane 1), coexpression of HDAC3 markedly increased ELL polyubiquitylation, as observed by immunoblotting using ubiquitin-specific antibody (compare lane 1 with lane 3). As expected, addition of MG132 further increased ELL polyubiquitylation (Fig. 5*B*, lane 4) and thus confirmed a role of HDAC3 in increasing ELL ubiquitylation. Subsequent analysis with increasing HDAC3 expression showed dose-dependent ELL destabilization (Fig. 5*C*, input lanes) and concomitant increased polyubiquitylation (Fig. 5*C*, lane 1 vs. lanes 3 to 6). This also correlates well with ELL acetylation wherein reduced acetylation shows concomitant increased polyubiquitylation (lane 1 vs. lanes 3 to 6). Therefore, we conclude that HDAC3-mediated ELL deacetylation promotes its polyubiquitylation and thus causes its degradation.

### DBC1 and p300 Increase ELL Acetylation and Decrease Its Poly-Ubiquitylation.

Based on our earlier observations (Figs. 3 and 4), we wondered whether both DBC1 and p300 would reduce HDAC3-mediated ELL polyubiquitylation. As shown in Fig. 5*D*, and consistent with our earlier observations, coexpression of DBC1 results in rescue of ELL destabilization by HDAC3 (input blot). Further IP analysis showed an increase in ELL acetylation and simultaneous reduced polyubiquitylation (Fig. 5*D*; compare lane 2 with lane 3). A similar experiment with p300 also showed rescue of HDAC3-mediated ELL degradation (Fig. 5*E*, input blots) that correlates well with its increased acetylation and decreased polyubiquitylation (Fig. 5*E*; compare lane 2 with lanes 3 to 5). Therefore, these results led us to conclude that p300-mediated ELL acetylation is protected from HDAC3-mediated deacetylation by DBC1 through its competitive binding to ELL. Increased ELL acetylation further causes decreased polyubiquitylation resulting in ELL stabilization.

### DBC1 and p300 Stabilize ELL against Ubiquitin-Mediated Degradation.

For addressing the correlation between acetylation and ubiquitylation of ELL, we initially wondered whether ELL protein would be degraded by increased expression of ubiquitin. Cotransfection analysis showed that, with overexpression of ubiquitin, the ectopically expressed ELL protein is also degraded (*SI Appendix*, Fig. S5 *A* and *B*, lane 5 vs. lane 3). Interestingly, overexpression of both DBC1 and p300 significantly rescued ubiquitin-mediated ELL degradation (*SI Appendix*, Fig. S5 *A* and *B*; compare lane 5 with lanes 6 to 8). Further IP analysis showed that both DBC1 and p300-mediated ELL stabilization (input lanes) strongly correlated with a concomitant increase in ELL acetylation as observed in *SI Appendix*, Fig. S5 *C* and *D* (compare lane 2 with lane 3 in both figures) that also strongly correlated with its decreased polyubiquitylation (*SI Appendix*, Fig. S5*C*, lane 2 vs. lane 3, and *SI Appendix*, Fig. S5*D*, lane 2 vs. lane 3). Therefore, based on these results, we conclude that both p300 and DBC1 increase ELL acetylation, which, in turn, stabilizes ELL protein through its reduced polyubiquitylation.

### N-Terminal K5 and K29 of ELL Are Key Targets for p300-Mediated Acetylation and Ubiquitin-Mediated Degradation.

Next, we addressed specific residue(s) of ELL that would be targeted for p300-mediated acetylation. Our initial in vitro acetylation of different purified ELL fragments (*SI Appendix*, Fig. S5 *E*, *Top*) by p300 showed that, upon deletion of the N-terminal 60 amino acids, p300-mediated ELL acetylation was significantly reduced (*SI Appendix*, Fig. S5 *E*, *Bottom*; compare lane 3 with lane 9 in both input GST blot and IP acetyl lysine blot). Further analysis showed the presence of only two key conserved lysine residues (K5 and K29) across multiple species (*SI Appendix*, Fig. S5*F*). Subsequent in vitro acetylation studies showed marked reduction in acetylation of the K5A, K29A mutant as compared to the wild type (WT) by p300 (*SI Appendix*, Fig. S5*G*; compare lanes 2 to 4 with lanes 6 to 8). Consistently, the ELL (K5A, K29A) mutant also showed reduced acetylation when coexpressed with p300 within mammalian cells (*SI Appendix*, Fig. S5*H*; compare lanes 2 to 3 with lanes 5 to 6). All of these analyses clearly show a role of ELL K5 and K29 residues as being key targets for p300-mediated acetylation both in vitro and in vivo within mammalian cells.

Next, we addressed whether these two key acetylation sites within ELL would also be targeted for ubiquitin-mediated degradation within mammalian cells. As shown in Fig. 5*F*, ELL fragments containing the N-terminal 45 amino acids are sensitive, whereas fragments losing those amino acids are completely resistant, to ubiquitin-mediated degradation (lanes 1 to 6 vs. lanes 7 to 12). Subsequent analysis using ELL (K5A, K29A) mutant construct showed complete resistance, whereas a parallel analysis using WT showed sensitivity to degradation by ubiquitin (Fig. 5*G*) and thus indicated that the N-terminal K5 and K29 of ELL are key targets for ubiquitin-mediated degradation. Our subsequent analysis also showed significant reduction in polyubiquitylation of ELL (K5A, K29A) mutant when compared to the WT (Fig. 5*H*, lanes 2 to 4 vs. lanes 6 to 8) in the presence of overexpressed ubiquitin. Consistent with a role for HDAC3-mediated deacetylation in increasing ELL ubiquitylation, coexpression of HDAC3 with ELL (K5A, K29A) mutant also showed significantly reduced ubiquitylation when compared to the WT (*SI Appendix*, Fig. S5*I*, compare lanes 2 to 4 with lanes 6 to 8). Further, overexpression of DBC1 also failed to show any increase in acetylation and resulting stabilization of ELL (K5A, K29A) mutant when compared to the WT (*SI Appendix*, Fig. S5*J*; compare lane 2 with lanes 4 to 6 and lane 8 with lanes 10 to 12). Thus, based on all of these results, we conclude that N-terminal K5 and K29 of ELL are key targets for p300-mediated acetylation and ubiquitin-mediated degradation of ELL. DBC1 and HDAC3 competitively bind to ELL and thus regulate ELL acetylation and its stability through modulating its polyubiquitylation. Although ELL (K5A, K29A) mutant showed enhanced stability, it showed decreased luciferase reporter gene activity compared with the WT (*SI Appendix*, Fig. S6*A*). This result further indicates additional role(s) of these two key residues in overall transcriptional regulation by ELL.

### E3 Ubiquitin Ligase Siah1-Mediated ELL Degradation Requires HDAC3.

Next, we were interested in identifying the E3 ubiquitin ligase that would be involved in ELL degradation within mammalian cells. An extensive literature survey showed that some of the SEC components, including ELL2 but not ELL, are targeted by Siah1 E3 ubiquitin ligase for their stability and regulation of target gene expression (14, 28). Consistently, we also failed to observe ELL degradation by Siah1 alone (Fig. 5*I*, lane 2 vs. lanes 3 to 5). We reasoned that, since degradation of ELL would also require its deacetylation by HDAC3, presence of HDAC3 is a prerequisite for Siah1-mediated degradation of ELL. As shown in Fig. 5*I*, in the presence of HDAC3, increased expression of Siah1 (*SI Appendix*, Fig. S6 *B*, *Lower*) resulted in increased degradation of ELL (lanes 3 to 5 vs. lanes 7 to 9) without affecting ELL mRNA expression (*SI Appendix*, Fig. S6 *B*, *Upper*). Thus, we conclude that human ELL protein is subjected to Siah1-mediated degradation only in the presence of HDAC3.

Based on all of these results, we conclude that, by targeting the same residues, p300-mediated acetylation causes stability of human ELL protein through reduced polyubiquitylation, whereas HDAC3-mediated deacetylation destabilizes ELL through increased polyubiquitylation. DBC1, by virtue of possessing the same binding site for ELL interaction, competes with HDAC3 and prevents ELL deacetylation and subsequent Siah1-mediated degradation (Fig. 5*J*).

### DBC1-Mediated ELL Stabilization Is Required for Expression of Diverse Sets of Genes.

Toward identifying the native target genes whose expression would be regulated by the newly identified DBC1−p300−HDAC3−Siah1−ELL axis, we initially intended to identify DBC1 and ELL coregulated genes. We noted two earlier studies that described an effect of ELL and DBC1 knockdown on global down-regulation of target genes in 293T cells (GSE34104 for ELL knockdown and GSE35480 for DBC1 knockdown) (29, 30). Considering the top 10,000 down-regulated genes, our analysis showed an overlap of a significant number of genes (∼37%) being co−down-regulated by both DBC1 and ELL (*SI Appendix*, Fig. S6*C*). Gene ontology (GO) analyses further showed a considerable fraction (24%) of these genes predicted to be involved in regulation of metabolic processes (*SI Appendix*, Fig. S6*D*). This is also consistent with the functional role of DBC1 that has been described by several studies until now (31).

**Fig. 6.**
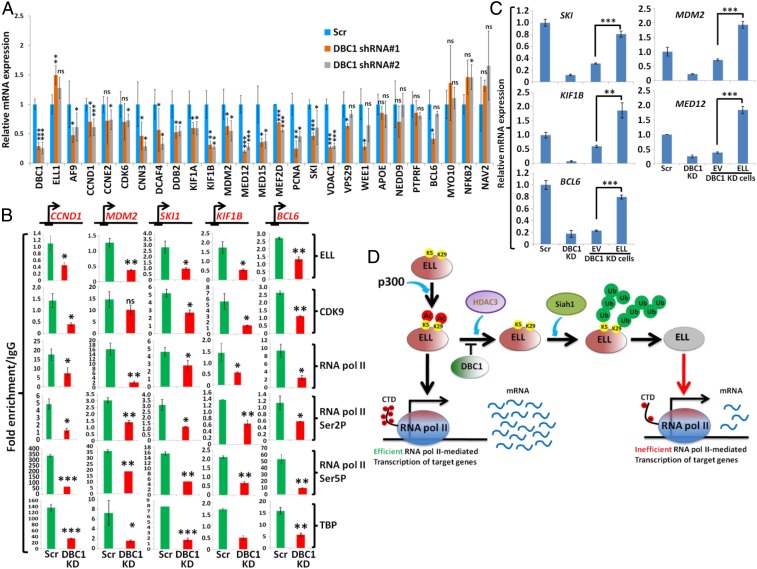
Down-regulation of target gene expression upon DBC1 knockdown is critically dependent on ELL. (*A*) The qRT-PCR analysis showing the effect of DBC1 knockdown, by using two different shRNAs, on basal level expression of target genes when compared to control scramble knockdown cells. (*B*) ChIP analysis showing the recruitment of target factors at the TSS region of indicated target genes upon DBC1 knockdown. (*C*) The qRT-PCR analysis showing the effect of reexpression of ELL on expression of selected target genes in DBC1 knockdown cells. (*D*) Cartoon diagram depicting mechanistic understanding of the role of DBC1, p300, HDAC3, and Siah1 in regulating ELL expression and downstream effect on expression of target genes. DBC1 knockdown results in simultaneous down-regulation of ELL by the action of HDAC3 deacetylase and Siah1-mediated polyubiquitylation. Reduced ELL level thus causes impaired expression of its target genes. In all of our statistical analyses, one-tailed Student’s *t* test was used to calculate the statistical significance of the data, wherein * denotes *P* ≤ 0.05, ** denotes *P* ≤ 0.01, *** denotes *P* ≤ 0.001, and ns denotes not significant.

For validating our high-throughput analysis using specific target genes, we generated two different stable DBC1 knockdown cell lines using shRNA constructs specific for DBC1 (*SI Appendix*, Fig. S6*E*). Interestingly, knockdown of DBC1 also resulted in decreased ELL protein level (*SI Appendix*, Fig. S6*E*) without affecting its RNA expression (Fig. 6*A*, ELL mRNA expression). Similar results were also observed on ELL protein level upon DBC1 knockdown in HCT116 cells (*SI Appendix*, Fig. S6*F*). Further RNA expression analysis showed that, upon DBC1 knockdown, the majority of selected target genes were down-regulated as shown in Fig. 6*A*. These target genes include key factors regulating cell proliferation (e.g., *CCND1*, *CCNE2*, *CDK6*, *PCNA*) as well as the basal transcription machinery (*MED12*, *MED15*). The effect of DBC1 on target gene expression is specific since the same knockdown cells did not show any effect on expression of nontarget genes such as *NEDD9*, *PTPRF*, *MYO10*, *NFκB2*, and *NAV2* (Fig. 6*A*). Consistent with the effect of DBC1 knockdown on reduced expression of cell proliferation-related genes, our subsequent assay showed reduced proliferation of 293T cells upon knockdown of DBC1 by two different shRNAs (*SI Appendix*, Fig. S7*A*).

Next, we addressed whether reduced ELL expression in DBC1 knockdown cells would result in its reduced recruitment, thus affecting downstream recruitment of SEC, on identified target genes. Our chromatin immunoprecipitation (ChIP) analyses showed reduced ELL recruitment at the transcription start site (TSS) on selected target genes upon DBC1 knockdown (Fig. 6*B*). Interestingly, and consistent with an earlier study showing a role of ELL in recruiting SEC components at the TSS region (29), we also observed significant down-regulation of recruitment of other SEC components, for example, CDK9. Further, ChIP analysis showed a strong reduction in Pol II recruitment as well as the presence of its Ser2 and Ser5 phosphorylated form, a hallmark of transcriptionally engaged elongating Pol II, on all target genes that we have tested upon DBC1 knockdown. The overall effect is target gene-specific since, in a parallel analysis, DBC1 knockdown failed to cause any impaired recruitment of all of the target factors on two nontarget *NEDD9* and *PTPRF* genes (*SI Appendix*, Fig. S6*G*) that we have tested. These results led us to conclude that, upon DBC1 knockdown, recruitment of SEC components along with Pol II at the TSS region of target genes are impaired (Fig. 6*B*).

Next, we addressed whether preinitiation complex (PIC) formation on target genes would also be impaired upon DBC1 knockdown. We performed TBP ChIP (as part of TFIID), as a candidate factor for PIC assembly, and also observed its impaired recruitment only on the target genes (Fig. 6*B*), but not on the two nontarget genes (*SI Appendix*, Fig. S6*G*). Consistent with their reduced recruitment, immunoblotting analysis confirmed reduced expression of the majority of TAF subunits of TFIID upon DBC1 knockdown (*SI Appendix*, Fig. S7*B*). Importantly, further analysis showed that RNA expression of a few TAF subunits, including TAF3 and TAF6, were impaired (*SI Appendix*, Fig. S7*C*). Since expression of TAF6 plays a key role in overall expression and assembly of other TAF subunits within TFIID (32), a down-regulation of TAF6 by DBC1 knockdown possibly results in overall impaired TFIID assembly and its recruitment and subsequent Pol II recruitment for target gene activation.

We next addressed whether the overall effect of DBC1 knockdown on transcriptional down-regulation of selected target genes is indeed a result of down-regulation of ELL expression, by reexpressing ELL in DBC1 knockdown cells. As shown in Fig. 6*C* and *SI Appendix*, Fig. S7*D*, while the empty vector failed to restore target gene expression, reexpression of ELL restored transcription in the majority of the genes that we have tested. Thus, we conclude that, upon DBC1 knockdown, simultaneous down-regulation of ELL results in transcriptional down-regulation of the target genes (Fig. 6*D*). Restoration of transcription of target genes by overexpression of ELL strongly suggests that ELL level is rate limiting in transcriptional down-regulation of target genes in DBC1 knockdown cells.

### Down-Regulation of Expression of DBC1, ELL, and Concomitant Glucose Homeostasis-Related Target Genes in Peripheral Blood Mononuclear Cells Isolated from Type 2 Diabetes Patients.

**Fig. 7.**
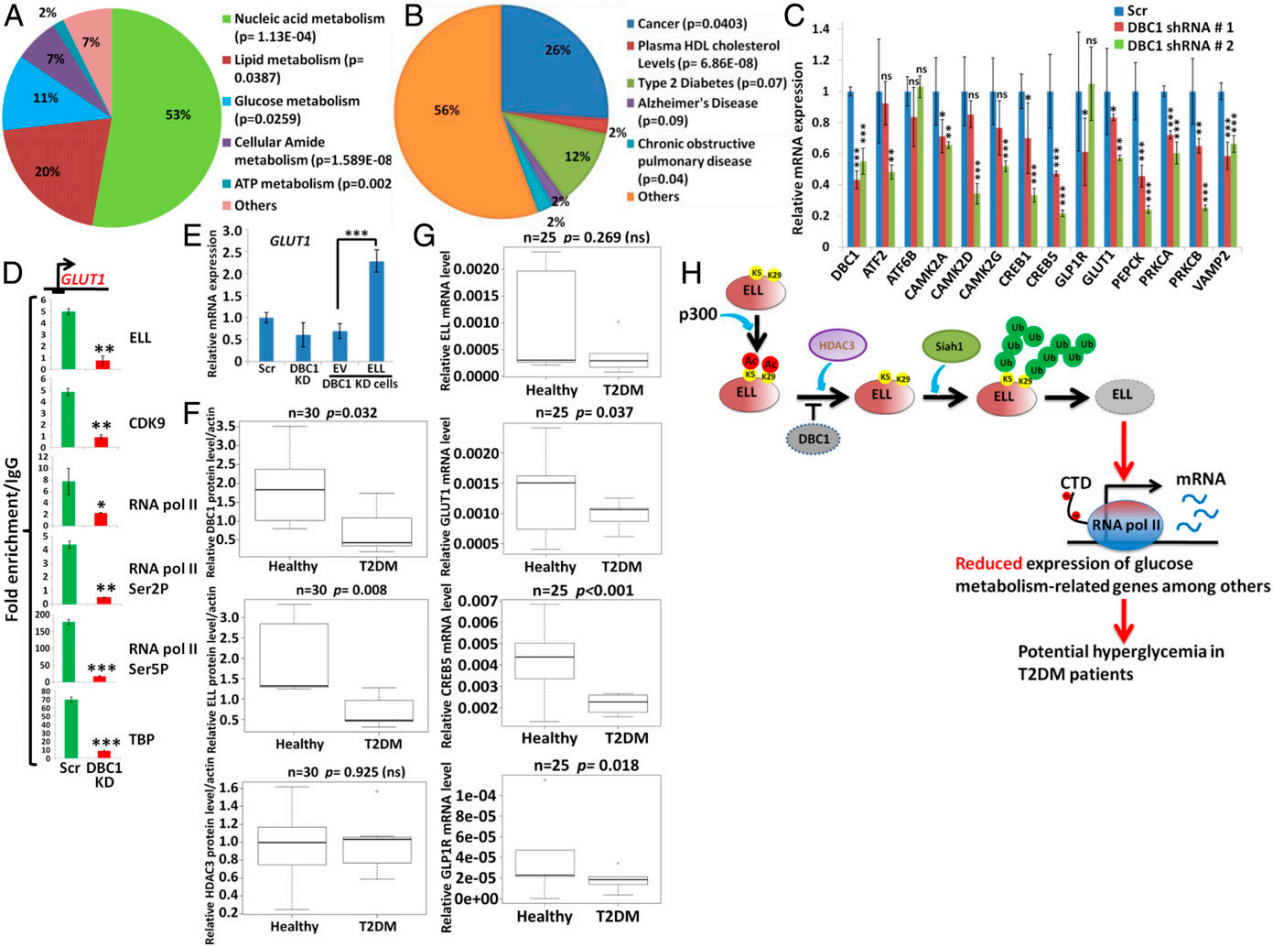
DBC1-mediated ELL stabilization is required for expression of genes that are associated with glucose homeostasis in humans. (*A*) GO analysis showing the different types of metabolic processes controlled by DBC1-ELL coregulated genes that are predicted to be involved in controlling metabolic processes (24% as shown in *SI Appendix*, Fig. S6*D*). (*B*) GO analysis predicting the involvement of DBC1-ELL coregulated genes (*SI Appendix*, Fig. S6*C*) in various human diseases. (*C*) The qRT-PCR analysis showing the effect of DBC1 knockdown on the basal level expression of various glucose metabolism-related genes compared to control scramble knockdown cells. (*D*) ChIP analysis showing the recruitment of several target factors at the TSS region of *GLUT1* gene upon knockdown of DBC1. (*E*) The qRT-PCR analysis showing the effect of reexpression of ELL in DBC1 knockdown cells on basal level expression of *GLUT1* gene in 293T cells. (*F*) Quantitation of the level of indicated target proteins (DBC1, ELL, and HDAC3) in healthy and T2DM patient cohort samples (*n* = 30 for each) as measured by Western blot analysis. Actin was used as loading control for each sample. (*G*) The qRT-PCR analysis showing the expression of different target genes (at RNA level) in healthy and patient cohort samples (*n* = 25). For the ease of dealing with total number of samples for subsequent qRT-PCR analysis, equal concentrations of RNA sample from five individuals were pooled (in an unbiased way) and mixed together to form a group. A total of five groups each of healthy and patient RNA samples were tested for presence of target RNAs. (*H*) Cartoon diagram showing the overall model of the role of p300-mediated acetylation, DBC1-mediated acetylation protection, HDAC3-mediated deacetylation, and subsequent Siah1-mediated degradation in maintaining ELL level for proper expression of target genes that are strongly associated with glucose homeostasis in healthy individuals. A down-regulation of upstream DBC1 in these processes leads to decreased ELL level through HDAC3-mediated degradation and thus possibly leads to decreased expression of key genes required for glucose homeostasis in healthy individuals, thus indicating a possible involvement of this mechanism of hyperglycemia in Type 2 diabetes patients. In all of our statistical analyses, one-tailed Student’s *t* test was used to calculate the statistical significance of the data, wherein * denotes *P* ≤ 0.05, **denotes *P* ≤ 0.01, *** denotes *P* ≤ 0.001, and ns denotes not significant.

Toward understanding a role for DBC1-mediated ELL stabilization in regulation of physiological processes, we further analyzed the set of metabolic genes that show down-regulation of expression upon knockdown of both DBC1 and ELL (*SI Appendix*, Fig. S6*D*). GO analysis predicted that a significant number of these genes are involved in metabolic processes, including nucleic acid, lipid, and glucose metabolism (Fig. 7*A*). Interestingly, the same analysis also predicted that misregulation of expression of a significant number of these coregulated genes (∼12%) is involved in pathogenesis of Type 2 diabetes (Fig. 7*B*). Consistent with this prediction, we observed significant down-regulation of a number of genes that are required for insulin secretion as well as glucose uptake pathways, including *GLUT1*, *ATF2*, and cAMP responsive proteins (*CREB1* and *CREB5*) upon DBC1 knockdown in 293T cells (Fig. 7*C*). Similar to our earlier results, we also observed impaired SEC as well as Pol II and TFIID recruitment at the TSS region of the *GLUT1* gene (Fig. 7*D*). Further, restoration of *GLUT1* gene expression through overexpression of ELL strongly indicated a role of ELL in the overall regulation of expression of *GLUT1* (Fig. 7*E*) in 293T cells. Thus, we conclude that DBC1-mediated ELL stabilization is required for proper expression of target genes that are strongly implicated in maintaining glucose metabolism in humans.

For further understanding a role for this pathway in glucose metabolism-related diseases, we noted a recent study that showed a strong association of transcriptional down-regulation of DBC1 and up-regulation of HDAC3 mRNA in peripheral blood mononuclear cells (PBMCs) of Type 2 diabetes patients (33) without providing any mechanistic insight. For understanding the underlying mechanisms, we collected PBMCs from a cohort of 30 control (healthy) and 30 treatment-naive patients and analyzed the level of target proteins being expressed in these samples. As shown in Fig. 7*F*, and consistent with the earlier study showing down-regulation at the RNA level (33), we also observed significant down-regulation of both DBC1 and ELL protein expression in the patient samples as compared to healthy controls (Fig. 7*F*). Interestingly, and consistent with our data in 293T cells, we failed to observe any significant down-regulation in ELL RNA level in the patient samples when compared to healthy controls (Fig. 7*G*, ELL panel). These data further indicated a key role of DBC1 in maintaining ELL protein level within patient samples. Contrary to the reported study showing up-regulation of HDAC3 RNA level in Type 2 diabetes patients (33), we did not observe significant changes in HDAC3 at the protein level. Thus, based on our mechanistic understanding from 293T cells, we conclude that the observed down-regulation of ELL protein level could as well be a result of down-regulation of DBC1 protein leading to increased ELL degradation by HDAC3 that remains unchanged between healthy and patient cohort samples.

Next, we asked whether DBC1 down-regulation and simultaneous reduced ELL level in patient PBMCs would also correlate with decreased expression of glucose metabolism-related genes as observed in 293T cells. As shown in Fig. 7*G*, RNAs extracted from patient samples showed significant down-regulation of expression of some of the key glucose uptake and metabolism-related genes such as *GLUT1*, *CREB5*, and *GLP1R*. Since these factors are well known for their role in maintaining glucose homeostasis in humans, a reduction of their expression upon reduced DBC1 expression could potentially lead to imbalance in cellular glucose level in the peripheral system as observed in our study.

Overall, our results in this study uncover important mechanistic insights into ELL stabilization within mammalian cells involving DBC1, p300, HDAC3, and E3 ubiquitin ligase, Siah1. Among these players, DBC1 provides an additional layer of stabilization to ELL by protecting its acetylation and thus increasing its stability. A down-regulation of DBC1 further leads to increased ELL deacetylation by HDAC3 and its subsequent degradation by Siah1. Reduced ELL levels contribute to decreased expression of diverse sets of genes, including the ones that are required for glucose homeostasis, in 293T cells. Strong correlation of these results with PBMCs isolated from Type 2 diabetes patients further indicates possible involvement of these mechanisms in overall disease pathogenesis (Fig. 7*H*). Further studies, using a large cohort of patients as well as animal models, would be required for in-depth understanding of the overall effect of reduced DBC1 and ELL expression and its implications in Type 2 diabetes pathogenesis.

## Discussion

In this study, we describe an important pathway in regulating the stability, and thus function, of a key SEC component, that is, ELL. Our results show a multilayered regulation involving DBC1, acetyl-transferase p300, deacetylase HDAC3, and an E3 ubiquitin ligase, Siah1. The overall mechanism of regulation is presented in Fig. 6*D*.

Two earlier studies described a role for human E3 ubiquitin ligase Siah1, but not Siah2, in regulating cellular levels of other SEC components such as ELL2 and AFF1 (14, 28). The study by Liu et al. (14) failed to observe ELL degradation by Siah1, whereas, in the same assay, ELL2 and AFF1 (to a less extent) showed extreme sensitivity. Our results provide a mechanistic explanation for their observation. We have clearly shown that ELL degradation by Siah1 requires concomitant HDAC3-mediated deacetylation. Thus, our study provides a paradigm for detailed understanding of the maintenance of cellular levels of all of these factors for proper ELL-mediated transcriptional response within mammalian cells during exposure to various environmental stimuli.

With our studies, we also show a role for DBC1, acting as an “acetylation protector” for ELL through competitive binding with HDAC3. Since the target for acetylation and Siah1-mediated polyubiquitylation happen to be on the same residues, interaction with DBC1 further protects ELL from HDAC3-mediated deacetylation and subsequent degradation by Siah1. This multiple layer of regulation of ELL, making it an extremely stable protein, is reflected in our CHX chase assay, wherein we have failed to observe any significant ELL degradation even after 24 h of chase (Fig. 2*G*). Besides ELL, DBC1 also showed interaction with other SEC components such as EAF1/2, CDK9, and AFF1 as evidenced by our IP analyses (Fig. 1*D*). Interestingly, DBC1 knockdown also reduced cellular levels of all of these proteins without affecting their mRNA expression (*SI Appendix*, Fig. S7 *E* and *F*). It would be interesting to further study whether DBC1 would also regulate abundance of other SEC components by the same mechanism as discussed in this study.

From our study, we also uncover an unexplored—yet interesting—role of ELL in regulation of expression of key genes that are required for maintaining several physiological processes, including glucose homeostasis. Notably, deregulation of a substantial number of genes that are coregulated by DBC1 and ELL has been predicted to be involved in giving rise to several diseases, including Type 2 diabetes (Fig. 7*B*). The strong correlation of effect of reduced level of DBC1 on expression of glucose homeostasis-related genes, both in 293T cells and PBMCs isolated from Type 2 diabetes patients, further indicates the possible importance of our study toward a mechanistic understanding of Type 2 diabetes pathogenesis. Nevertheless, these interesting observations have to be studied in detail for a deeper mechanistic understanding of the role of these mechanisms in overall Type 2 diabetes pathogenesis.

## Materials and Methods

Details of all materials regarding list of plasmids, primers for RNA and ChIP analyses, and antibodies used for our studies can be found in *SI Appendix*. Further, methods detailing cell culture techniques, creation of different plasmid constructs, stable cell line generation, mass spectrometry analysis, nuclear extract preparation, IP analysis, protein complex purification from nuclear extract, luciferase assay, CHX chase assay, recombinant protein purification, in vitro acetylation assay, in vitro deacetylation assay, GO analysis, ChIP analysis, RNA analysis using qRT-PCR, and PBMC isolation can also be found in *SI Appendix*.

### Clinical Studies and Collection of Blood Samples.

A total of 78 individuals were recruited from the IPGME&R, Kolkata, following the American Diabetes Association criteria (34) for the clinical studies described. Of these, 34 were treatment-naïve Type 2 diabetes mellitus patients, and 32 were healthy, age-matched controls. The research study was approved by the Institutional Ethics and Research Committee of IPGME&R and CSIR-IICB, and all test subjects provided informed consent. Blood samples were collected from patients with Type2 diabetes and age- and gender-matched healthy controls after obtaining written informed consent from each study participant in his or her own vernacular.

### Data Availability.

All of the data for this study are included in the manuscript and *SI Appendix*.

## Supplementary Material

Supplementary File
