# Supplementary material for: DBC1, p300, HDAC3, and Siah1 coordinately regulate ELL stability and function for expression of its target genes
Source: Proc Natl Acad Sci U S A. 2020 Mar 9;117(12):6509–20. doi: 10.1073/pnas.1912375117 (PMC7104407; doi:10.1073/pnas.1912375117)
Supplement: Supplementary File [file pnas.1912375117.sapp.pdf]

SI Appendix

A

Name of Protein	Percentage coverage	no of unique peptides	Total no of peptides	Representing peptides	
KIAA 1967	42.6	34	62	AAEAAPPTQEAQGETEPTEQAPDALEQAADTSR	
KIAA 1967	42.6	34	62	KEEEAVLVGGEWSPSLDGLDPQADPQVLVR	
KIAA 1967	42.6	34	62	EAAPDAGAEPITADSDPAYSSK	
KIAA 1967	42.6	34	62	AAYNPGQAVPWNAVK	
KIAA 1967	42.6	34	62	SPAPPLLHVAALGQK	
KIAA 1967	42.6	34	62	FAEFQYLQPGPPR	
KIAA 1967	42.6	34	62	EAAPDAGAEPITADSDPAYSSK	

B

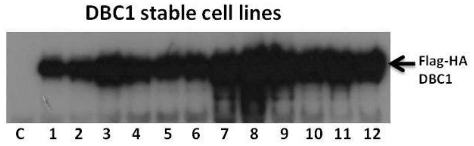

C

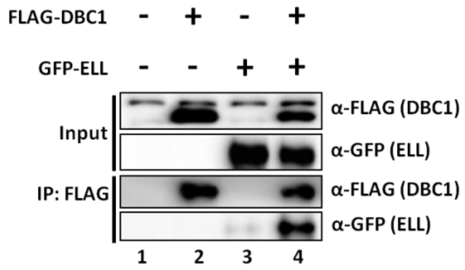

Fig. S1: Identification of DBC1 as ELL interacting proteins

A. Mass Spectrometric analysis showing DBC1 peptides in our ELL.com preparation as discussed earlier (1).

B. Western blotting analysis showing generation of stable DBC1 cell line ectopically expressing DBC1 as FLAG-HA-tagged. Lane C denotes control in this experiment.

C. Co-IP and subsequent western blotting analysis showing interaction between DBC1 and ELL through their co-expression in 293T cells.

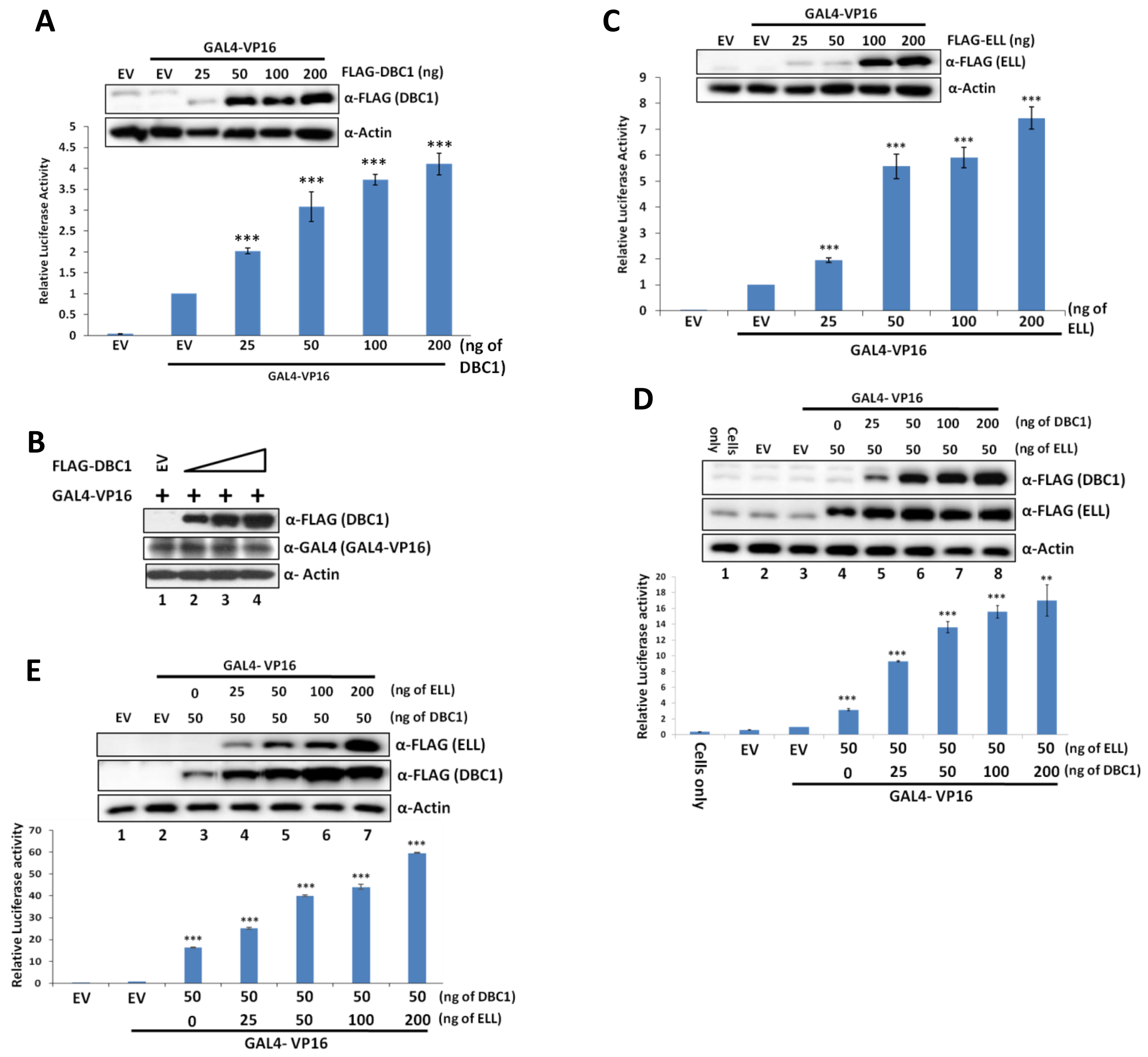

Fig. S2: Positive regulation of transcription of chromosomally integrated reporter gene by DBC1 and ELL

A. Effect of DBC1 on activator (GAL4-VP16)-dependent chromosomally integrated luciferase reporter gene expression. The inset panel shows expression of transfected DBC1 within Gal-luciferase cells.

B. Western blot analysis showing effect of increasing expression of DBC1 on expression of activator GAL4-VP16 within Gal-luciferase cells.

C. Effect of ELL on activator (GAL4-VP16)-dependent chromosomally integrated luciferase reporter gene expression. The inset panel shows expression of transfected ELL in the Gal-luciferase cells.

D. Effect of increasing expression of DBC1 with constant ELL expression on activator (GAL4-VP16)-dependent reporter gene expression. The inset panel shows expression of transfected ELL and DBC1 in the Gal-luciferase cells.

E. Effect of increasing expression of ELL with constant DBC1 expression on activator (GAL4-VP16)-dependent reporter gene expression. The inset panel shows expression of transfected ELL and DBC1 in the Gal-luciferase cells.

In all the experiments mentioned above, one-tailed student's t test was used to calculate the statistical significance of the data. * denotes p≤0.05, **denotes p≤0.01, *** denotes p≤0.001, and ns denotes not significant.

.

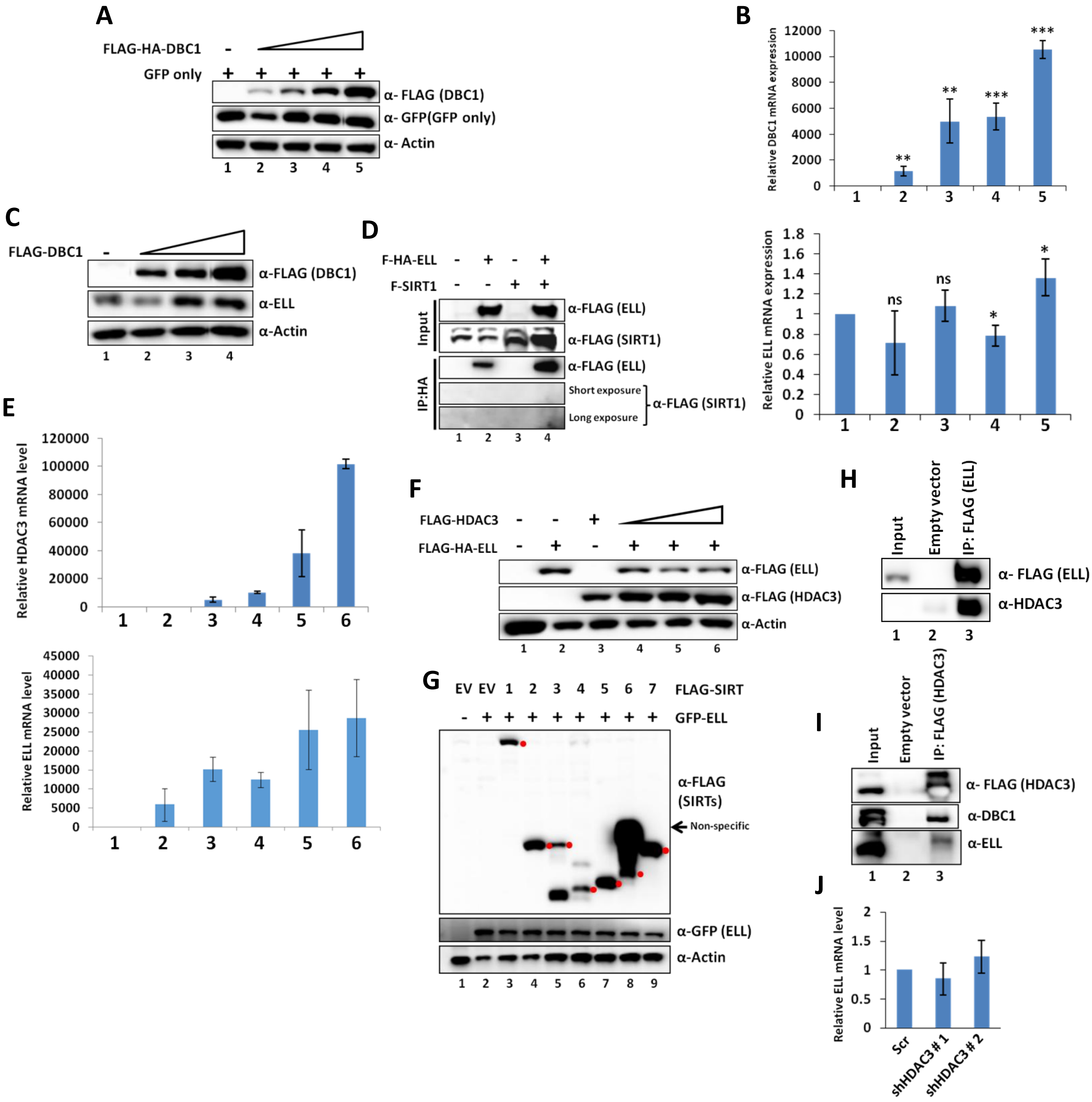

Fig. S3: Interaction of various deacetylases with ELL and their effect on regulating ELL stability

A. Western blot analysis showing the effect of DBC1 over-expression on ectopic expression of control GFP.

B. qRT-PCR analysis showing levels of DBC1 (top panel) and ELL (bottom panel) mRNA (normalized to 18s rRNA) when increasing amount of DBC1 is co-expressed with constant ELL (Fig. 2A). For showing relative RNA level, 18s rRNA normalized values were further normalized to the value in lane no. 1 for each of the DBC1 and ELL RNA levels.

C. Western blot analysis showing the effect of over-expression of DBC1 on endogenous ELL expression in HCT116 cells..

D. Co-IP and western blotting analysis showing absence of ELL and SIRT1 interaction in 293T cells.

E. qRT-PCR analysis showing ELL (lower panel) and HDAC3 (upper panel) mRNA (relative to 18s rRNA) level in presence of over-expressed HDAC3 (Fig. 2E).

F. Western blot analysis showing the effect of over-expression of HDAC3 on ectopic expression of ELL in HCT116 cells

G. Western blot analysis showing the effect of over-expression of SIRT1-7 on ELL stabilization in 293T cells. Red filled circles indicate target protein bands in our assay. Other bands present on the blot are either degradation or non-specific in nature. In presence of over-expressed SIRT6, we consistently observe the appearance of additional bands above the target SIRT6 band when immunoblotted with α-FLAG antibody.

H. Western blotting showing interaction of ELL and HDAC3 in 293T cells by immunoprecipitation of ELL.

I. Western blotting showing interaction of HDAC3 and ELL in 293T cells by immunoprecipitation of HDAC3. Presence of DBC1 was used as a positive control in this assay.

J. Effect of HDAC3 knockdown on ELL mRNA level as measured by qRT-PCR analysis in 293T cells.

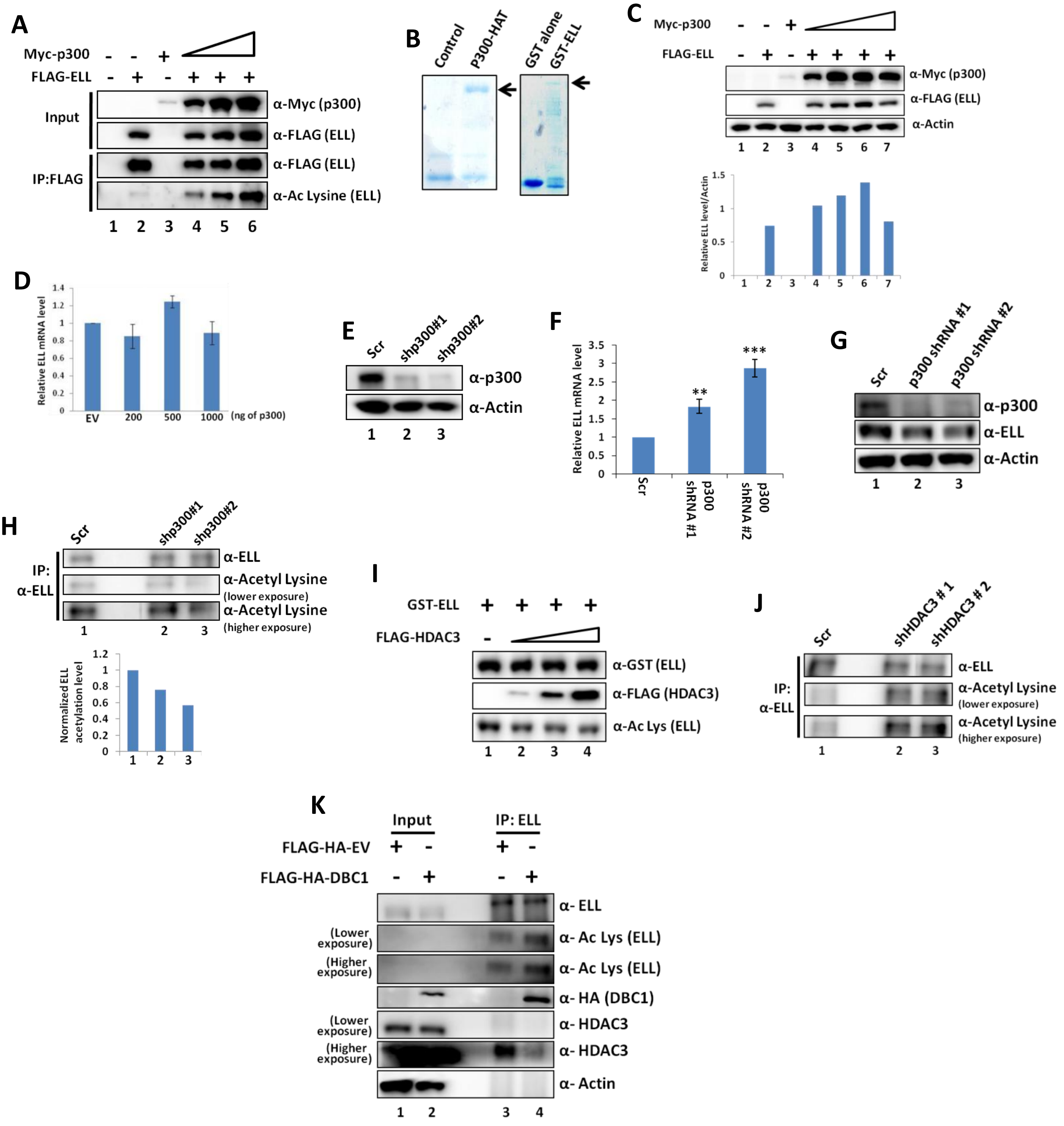

Fig. S4: p300-mediated acetylation stabilizes ELL

A. Western blot analysis showing ELL acetylation through increased over-expression of p300 within 293T cells.

B. Purification of the HAT domain of p300 and GST-ELL (using bacterial expression system) that were used for *in vitro* acetylation of ELL by p300 (Fig. 4B).

C. Western blot analysis showing the effect of increasing over-expression of p300 on ectopic expression of ELL in 293T cells (upper panel) and quantitation of ELL protein relative to Actin (lower panel).

D. qRT-PCR analysis showing the effect of p300 over-expression on expression of endogenous ELL mRNA (Fig. 4C) normalized to 18s rRNA. The data obtained were further normalized with value in EV control lane.

E. Western blot analysis showing stable p300 knockdown by two different shRNAs within 293T cells.
F. RNA analysis showing mRNA levels of endogenous ELL in the two generated p300 knockdown cell lines when compared to 18s rRNA. The data obtained were further normalized with the control scramble sample.
G. Western blot analysis showing effect of stable p300 knockdown, by two different shRNA constructs, on endogenous ELL protein level within HCT116 cells.
H. Immunoprecipitation and western blotting analysis showing the effect of p300 knockdown on acetylation levels of endogenous ELL. The bottom panel shows the quantification of acetylation signal as compared to that of scramble control cells.
I. *In vitro* deacetylation assay to show deacetylation of ELL by purified HDAC3.
J. Immunoblots showing the acetylation status of endogenous ELL in HDAC3 knockdown cell lines.
K. Immunoprecipitation of endogenous ELL in control and DBC1 over-expressed 293T cells and subsequent western blotting analysis showing decreased association of endogenous ELL with HDAC3 upon its binding with ectopic DBC1. Interestingly, the reduced HDAC3 binding upon DBC1 over-expression also results in increased acetylation of endogenous ELL as can be seen in the IP panel.

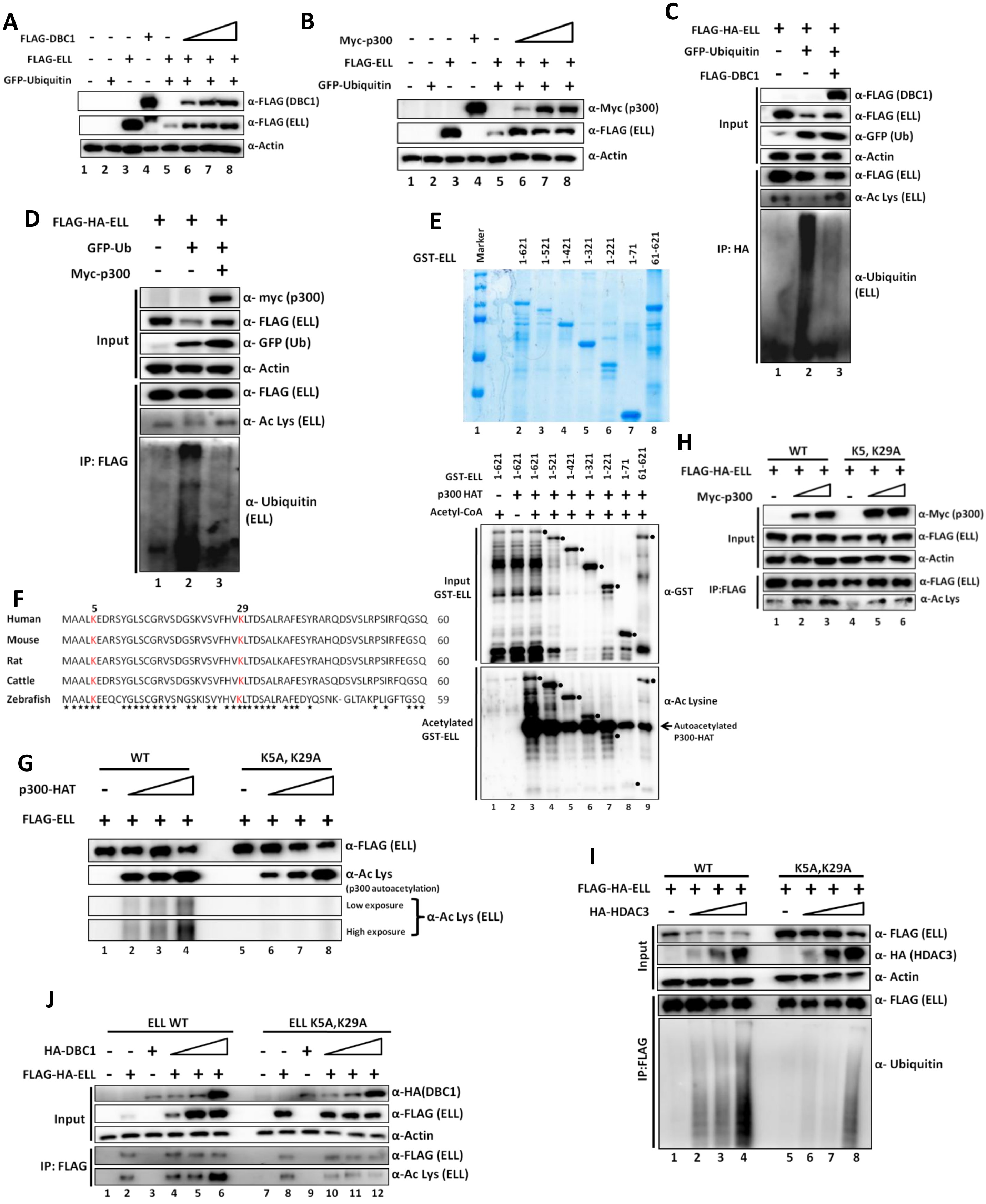
A
FLAG-DBC1
FLAG-ELL
GFP-Ubiquitin
α-FLAG (DBC1)
α-FLAG (ELL)
α-Actin
1 2 3 4 5 6 7 8
B
Myc-p300
FLAG-ELL
GFP-Ubiquitin
α-Myc (p300)
α-FLAG (ELL)
α-Actin
1 2 3 4 5 6 7 8
C
FLAG-HA-ELL
GFP-Ubiquitin
FLAG-DBC1
Input
α-FLAG (DBC1)
α-FLAG (ELL)
α-GFP (Ub)
α-Actin
IP: HA
α-FLAG (ELL)
α-Ac Lys (ELL)
α-Ubiquitin (ELL)
1 2 3
D
FLAG-HA-ELL
GFP-Ub
Myc-p300
Input
α- myc (p300)
α- FLAG (ELL)
α- GFP (Ub)
α- Actin
IP: FLAG
α- FLAG (ELL)
α- Ac Lys (ELL)
α- Ubiquitin (ELL)
1 2 3
E
GST-ELL
Marker
1-621
1-521
1-421
1-321
1-221
1-71
61-621
1 2 3 4 5 6 7 8
GST-ELL
p300 HAT
Acetyl-CoA
1-621
1-621
1-621
1-521
1-421
1-321
1-221
1-71
61-621
Input GST-ELL
α-GST
Acetylated GST-ELL
α-Ac Lysine
Autoacetylated P300-HAT
1 2 3 4 5 6 7 8 9
F
5
29
Human MAALKEDRSYGLSCGRVSDGSKVSVFHVKLTDSALRAFESYRARQDSVSLRPSIRFQGSQ 60
Mouse MAALKEARSYGLSCGRVSDGSRVSVFHVKLTDSALKAFESYRAHQDSVSLRPSIRFEGSQ 60
Rat MAALKEARSYGLSCGRVSDGSRVSVFHVKLTDSALKAFESYRAHQDSVSLRPSIRFEGSQ 60
Cattle MAALKEDRSYGLSCGRVSDGSKVSVFHVKLTDSALRAFESYRASQDSVSLRPSIRFQGSQ 60
Zebrafish MAALKEEQCYGLSCGRVSNGSKISVYHVKLTDSALRAFEDYQSNK- GLTAKPLIGFTGSQ 59
G
WT
K5A, K29A
p300-HAT
FLAG-ELL
α-FLAG (ELL)
α-Ac Lys
(p300 autoacetylation)
Low exposure
High exposure
α-Ac Lys (ELL)
1 2 3 4 5 6 7 8
H
WT
K5, K29A
FLAG-HA-ELL
Myc-p300
Input
α-Myc (p300)
α-FLAG (ELL)
α-Actin
IP:FLAG
α-FLAG (ELL)
α-Ac Lys
1 2 3 4 5 6
I
WT
K5A,K29A
FLAG-HA-ELL
HA-HDAC3
Input
α- FLAG (ELL)
α- HA (HDAC3)
α- Actin
IP:FLAG
α- FLAG (ELL)
α- Ubiquitin
1 2 3 4 5 6 7 8
J
ELL WT
ELL K5A,K29A
HA-DBC1
FLAG-HA-ELL
Input
α-HA(DBC1)
α-FLAG (ELL)
α-Actin
IP: FLAG
α-FLAG (ELL)
α-Ac Lys (ELL)
1 2 3 4 5 6 7 8 9 10 11 12

Fig. S5: Over-expression of DBC1 and p300 stabilizes ELL from ubiquitin-mediated degradation.

A. Western blot analysis showing the rescue of ubiquitin-mediated ELL degradation through concomitant over-expression of DBC1.

B. Western blot analysis showing the rescue of ubiquitin-mediated ELL degradation through concomitant over-expression of p300.

C. Western blot analysis showing a strong correlation of increase in acetylation and decrease in ubiquitin mediated poly-ubiquitylation of ELL through concomitant over-expression of DBC1 in 293T cells.

D. Western blot analysis showing strong correlation of increase in acetylation and decrease in ubiquitin-mediated poly-ubiquitylation of ELL through concomitant over-expression of p300 in 293T cells.

E. Coomassie staining of purified GST-tagged full-length and deletion mutants of ELL (as indicated) (top panel). In vitro acetylation assay and subsequent immunoblotting showing acetylation of different ELL fragments by p300 HAT (bottom panel). The blots were also probed with α-GST antibody to show the amount of GST-ELL protein used in our *in vitro* assays.

F. Multiple sequence alignment of the N-terminal 60 amino acids of ELL showing conserved nature of Lysine 5 (K5) and Lysine 29 (K29) across various species.

G. *In vitro* acetylation assay showing p300-mediated acetylation of ELL (WT) and ELL(K5A, K29A) mutant.

H. Western blot analysis showing defective acetylation of ELL (K5A, K29A) mutant as compared to wild type in presence of over-expressed p300 within 293T cells.

I. Western blotting analysis showing HDAC3-mediated poly-ubiquitylation of ELL (WT) and ELL (K5A, K29A) mutant within 293T cells.

J. Western blotting analysis showing an effect of DBC1 over-expression on expression of ELL (WT) and K5A, K29A mutant in 293T cells (input panels). Further, consistent with a role of K5 and K29 sites ELL as targets for acetylation, DBC1 over-expression fails to show any increase in acetylation in ELL (K5A, K29A) mutant when compared to the wild type.

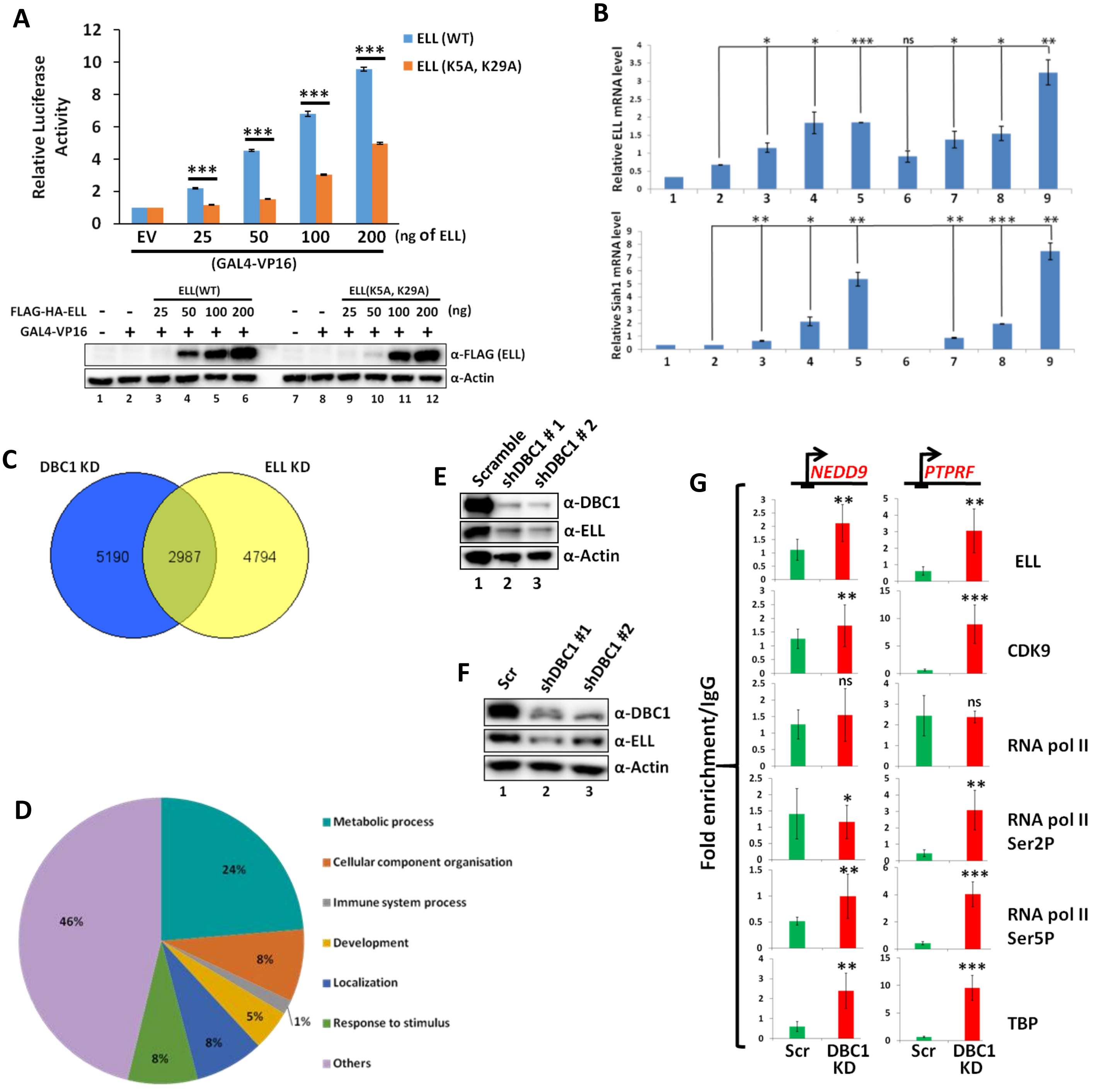

Fig. S6: Global co-regulation of gene expression by DBC1 and ELL are implicated in multiple cellular events

A. Reporter assay showing the effect of ELL (WT) and ELL (K5A, K29A) on activator (GAL4-VP16)-dependent chromosomally-integrated luciferase reporter gene (upper panel). The lower panel shows the expression levels of the ELL constructs used in the assay.

B. qRT-PCR showing Siah1 (lower panel) and ELL (upper panel) mRNA expression in the experiment as described in Fig. 5I. Interestingly, in presence of increased Siah1 expression, ELL mRNA level does not decrease thus indicating an effect of Siah1 on decreased ELL protein level is a result of its effect on ELL protein and not RNA.

C. Venn Diagram showing the number of overlapping genes that are down-regulated by knockdown of both DBC1 (GSE35480) and ELL (GSE34104) in 293T cells as obtained from two different publicly available datasets.

D. Pathway analysis predicting cellular processes that are affected by DBC1-ELL co-regulated genes.

E. Western blotting analysis showing stable knockdown of DBC1 in 293T cells by two different shRNAs. Decreased DBC1 also results in a concomitant decrease in ELL protein level.

F. Immunoblots showing stable knockdown of DBC1 in HCT116 cells by two different shRNAs. Here also, decreased DBC1 also results in concomitant decrease in ELL protein level.

G. ChIP analysis showing the recruitment of indicated target factors at the TSS of *NEDD9* and *PTPRF* genes, whose expressions are not impaired upon DBC1 knockdown as shown in Fig. 6A.

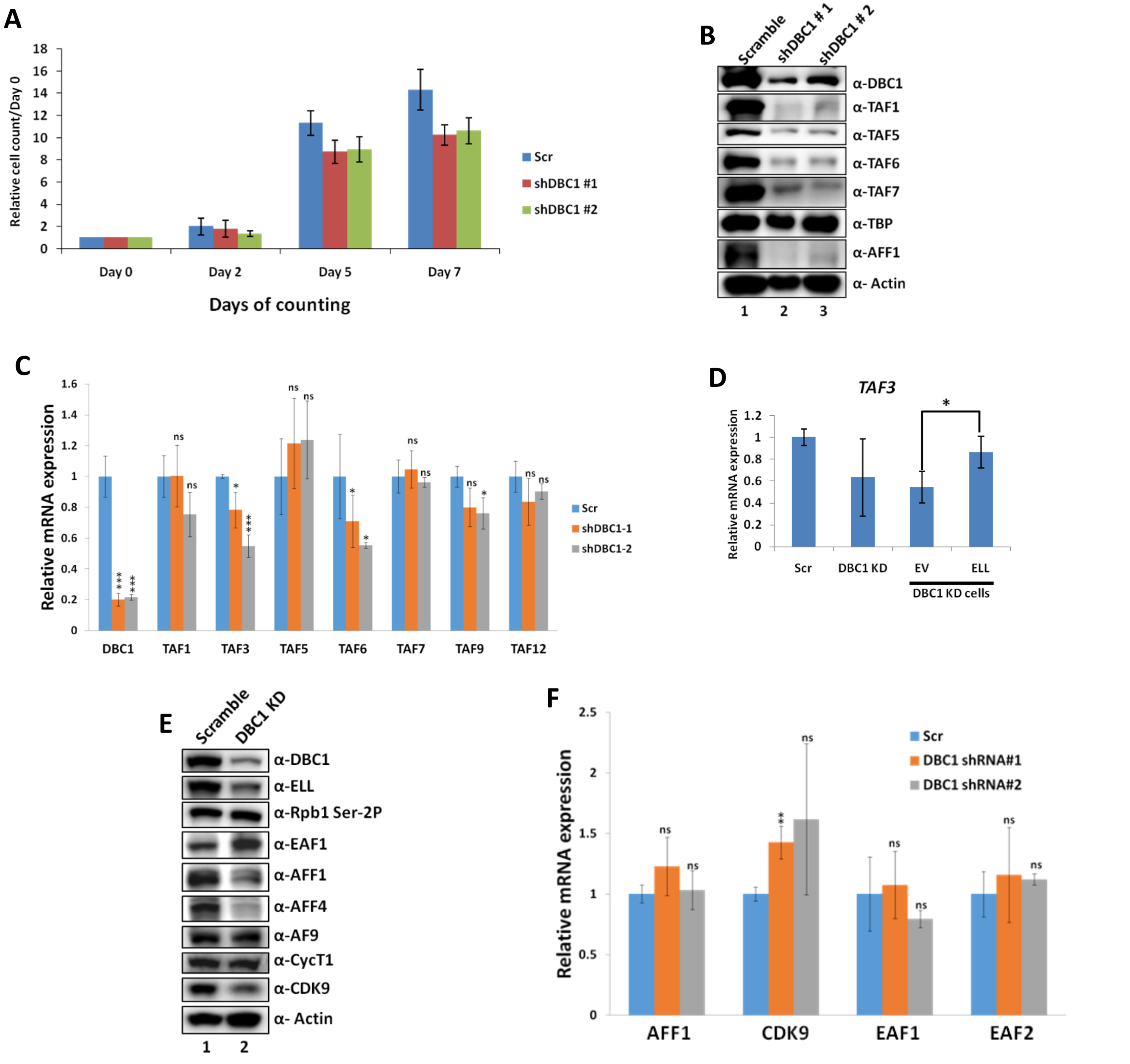

Fig. S7: Knockdown of DBC1 causes down-regulation of expression of TAF components of TFIID as well as key SEC components.

A. Cell proliferation assay demonstrating an effect of DBC1 knockdown (by two different shRNAs) on proliferation of 293T cells. Equal number of cells were seeded and counting was done on the indicated days.

B. Western blot analysis showing the effect of DBC1 knockdown on the expression of target TAF subunits of TFIID in 293T cells.

C. qRT-PCR analysis showing the effect of DBC1 knockdown on mRNA expression of indicated TAF subunits of TFIID.

D. qRT-PCR analysis showing the effect of re-expression of ELL in DBC1 knockdown cells on the restoration of expression of TAF3 subunit of TFIID.

E. Western blot analysis showing the effect of DBC1 knockdown on the expression of SEC subunits in 293T cells.

F. qRT-PCR analysis showing effect of DBC1 knockdown on the expression of indicated SEC subunits at the mRNA level.

In all of our statistical analyses, one-tailed student's t test was used to calculate the statistical significance of the data, wherein, * denotes $p \leq 0.05$, **denotes $p \leq 0.01$, *** denotes $p \leq 0.001$, and ns denotes not significant.

Materials and Methods:

Supplemental Table 1: List of Plasmids used in this study

Name of plasmid	Plasmid details	Source
M10	pcDNA5/FRT/TO vector with cloned FLAG-HA tag	Biswas et al., 2011
M15	ELL cloned into M10 vector (Biswas et al., 2011)	Biswas et al., 2011
M43	pFASTBAC vector	Invitrogen
M44	pFASTBAC vector with FLAG tag	Biswas et al., 2011
M56	pGEX vector with GST tag	Amersham
M61	ELL cloned into M56 vector	This study
M90	6-His pET-11d vector	Kumari et al., 2019
M296	psPAX2 lentiviral packaging plasmid	Addgene #12260
M297	pMD2.G VSV-G envelope plasmid	Addgene #12259
M336	DBC1 cloned into M10 vector	This study
M337	DBC1 cloned into M44 vector	This study
M340	pM vector	Clontech
M378	FLAG-PCAF cloned in pCI vector	Addgene #8941
M379	HA-CBP in pRc/RSV vector	Addgene #16701
M380	FLAG-HDAC1 in pcDNA3.1	Addgene #13820
M381	FLAG-SIRT1 in pECE vector	Addgene #1791
M442	VP16 Activation domain cloned into M340 vector	Ghosh et al., 2018
M447	pcDNA5/FRT/TO vector with cloned FLAG tag	Yadav et al., 2019
M451	pcDNA5/FRT/TO vector with cloned myc tag	This study
M464	DBC1 cloned into M447 vector	This study
M496	pEGFP-N2 vector	Clontech
M498	ELL cloned into M496	This study
M532	P300 cloned in M451	Kumari et al., 2019
M594	Myc tagged DBc1 in pcDNA3	Addgene #35096
M686	p300 HAT domain (1284-1672 aa) cloned in M90	Kumari et al., 2019
M742	ELL (1-500 aa) cloned into M447 vector	This study
M743	ELL (1-373 aa) cloned into M447 vector	This study
M744	ELL (45-621 aa) cloned into M447 vector	This study
M745	ELL (374-621 aa) cloned into M447 vector	This study
M746	ELL (45-373 aa) cloned into M447 vector	This study
M747	ELL cloned into M447 vector	This study
M773	MOF cloned in M449 vector	Kumari et al., 2019
M787	FLAG-HDAC4 in pcDNA3 vector	Addgene # 30485
M788	FLAG-HDAC7 in pcDNA3.1 vector	Addgene 13824
M789	FLAG-HDAC8 in pcDNA3.1 vector	Addgene # 13825
M790	FLAG-HDAC2 in pcDNA3 vector	Addgene # 68117
M791	FLAG-HDAC6 in pcDNA3.1 vector	Addgene # 13823
M792	FLAG-HDAC3 in pcDNA3.1 vector	Addgene #13819
M794	FLAG-GCN5 in pAdEasy vector	Addgene #14106
M796	FLAG-HDAC5in pcDNA3.1 vector	Addgene #13822
M912	Ubiquitin cloned in EGFP-C1 vector	Addgene #11928

M987	ELL (K5A) mutant cloned in M10 vector	This study
M1006	ELL (K5A, K29A) mutant cloned in M10 vector	This study
M1013	SIAH1 cloned into M10 vector	This study

Supplemental Table 2: List of antibodies used in this study

Antibodies	Source
ELL	Bethyl Laboratories and Cell Signaling Technology
EAF1	Santa Cruz Biotechnology
EAF2	Abcam
CCNT1	Santa Cruz Biotechnology
CDK9	Santa Cruz Biotechnology
DBC1	Bethyl Laboratories and Cell Signaling Technology
AF4	Abcam
TBP	Cell Signaling Technology
AF9	Bethyl Laboratories
β-Actin	Santa Cruz Biotechnology
P300	Cell Signaling Technology
Rpb1 CTD (4H8 clone)	Cell Signaling Technology
Ubiquitin	Cell Signaling Technology
Flag epitope	Sigma
HA epitope	Santa Cruz Biotechnology
His epitope	SantaCruz Biotechnology
Myc epitope	Cell Signaling Technology
GST	Santa Cruz Biotechnology
GFP	BioBharati Life Science
Acetylated Lysine	Cell Signaling Technology
Phospho Rpb1 CTD (Ser2)	Cell Signaling Technology
Phospho Rpb1 CTD (Ser5)	Cell Signaling Technology
Normal Rabbit IgG	Cell Signaling Technology
Normal Mouse IgG	Cell Signaling Technology
HRP conjugated secondary antibody (Rabbit)	Bio-Rad
HRP conjugated secondary antibody (Mouse)	Cell Signaling Technology

Supplemental Table 3: List of qRT-PCR primers used for RNA analysis in this study

Gene	Forward Sequence	Reverse Sequence
AF9	GAGAGAAAGACACATTCTGC	GGATGTTCCAGATGTTTCC
APOE	GCGGATGGAGGAGATG	CTCGAACCAGCTCTTG
ATF2	GTACCAGGCCCATTTCCTCTTC	GAACGAGTGGGACTGCAGCTG

ATF6B	CATCGTTCCCGCTCCTATGCCT	CTTCTTTCTCCGGGACTGGCAG
BCL6	CCACACAGGAGAGAAACCTTAC	GCAGGTCAGTGGCTGACAC
CAMK2A	GAAGTGCTGCGGAAGGACCC	CAGGCGGTGCTGGTCCTCATC
CAMK2D	TATCAACAATGGGGACTTTG	GTAGAATCGGTGAAAATCC
CAMK2G	GATGAGGATCAGCACAAGCTG	GGTCAGCATCTGGTTGATCAAG
CCND1	TCTAAGATGAAGGAGACCAT	GGAAGTGTTCAATGAAATCG
CCNE2	GCTGGCCTATGTGACTTACC	GGTAGACTTCGATGGGCCC
CDK6	GGAGTGTTGGCTGCATATTTG	CGATATCTGTTACAAACTTC
CNN3	CTACAGAACCTGTCATTC	ATCTCTGGGGTAGTCATC
CREB1	GATTCACAGGAGTCAGTGGATAG	GCACTCCTGGTGCATCAGAAG
CREB5	GAAGGCTGCATTGACTCACCAC	GTGCATGTGATGGTGTGGCGTC
DBC1	GAGGAGTTTGCAGGAGC	GTAGCCACACCAGTTGG
DCAF4	AAACCTCTACTTCACCAACC	CTGGGTGACTATTGACGAA
DDB2	TACCCCTTATGAATTGAGG	CAGATGAGAATGTGGTAAC
ELL	GACCAACACCAACTACAGCCAGG	GTACTCGGCGATGAGCCTCTTG
GLP1R	GCATCCTTCATCCTGCGAGCAT	GCATGAGCAGAAACACCAGGCG
GLUT1	GAAGTGTCACCCACAGCCCTTC	GGGCCACAGGTCCTTGTTGC
KIF1A	AGAAGGTGAGCAAAATCAG	CGGAATGAAATCTGTCTTC
KIF1B	AATCAGAGTGACTTTTCGTC	ATTCGACTGATTTCTTCTGG
MDM2	TTGGATCAGGATTCAGTTTC	GAGAGTTCTTGTCCTTCTTC
MED12	CAGGATGAACAACGCGAG	GAGTGCCTCATGCATAAGC
MED15	GACCTTTAAACACACCTGTG	GGCTCTTCATCTTACTCAGG
MEF2D	GACCTGAACAGTGCTAACG	GTGATGACTCGCAGGTC
MYO10	CTTCAGGATGAGGCCATC	GCCAGCTGTACAGGTTG
NAV2	GTAATTAACACACCTGAGC	TCTTCTTTGAGTCACTCTC
NEDD9	CCCATCCAGATACCAAAAGGAC	GTAGGCGGGATGTCATACAC
NFKB2	GAGAACGGAGACACACC	CAGAAAGCTCACCACAC
PEPCK	GGTACCTCTGCCACCACCAATC	CACCAGGTGGAAGAGGCTGGTC
PCNA	GGAGGAAGCTGTTACCATAGAG	CCTCGATCTTGGGAGCCAAG
PRKCA	CAGGAAGGGCACAGAAGAACTG	GAACGGGGGTTTGTCAAGCAG
PRKCB	GAATTGGAAGGTTCAGTGAGCC	CCTGGATATAGCCTTGATGGTC
PTPRF	CAATGCCAGCTTCCTGGATG	ATGATGGTGGAATTGTGCTC
SKI	CCAAGTACTCGGCCCAGATC	GCACGGAATCTACGGCTCC
TAF6	CTTGCTGAAGGTTCCTG	GAAGTGGTGGTGGAAC
VDAC1	CAAGTATCAGATTGACCCTG	GTCAGTTTAATACCTGGC
VPS29	GCATATAATGCCTTGGAAAC	CACATCATCTCCAATTAGC
WEE1	GCTGTCCGCTTCTAGAAAG	CGAGATGTTCTATTACTCTGGG
18s rRNA	GTAACCCGTTGAACCCCATT	CCATCCAATCGGTAGTAGCG

Supplemental Table 4: Oligo Sequences for shRNA construct generation for DBC1 knockdown

Gene	Upper Oligo	Lower Oligo
DBC1 sh#1	CCGGGCCAAAGGAAAGGATCTCTTTCTCG AGAAAGAGATCCTTTCCTTTGGCTTTTTG	AATTCAAAAAGCCAAAGGAAAGGATCTCTTTCTCGAGAA AGAGATCCTTTCCTTTGGC
DBC1	CCGGGCATTGATTTGAGCGGCTGTACTCG	AATTCAAAAAGCATTGATTTGAGCGGCTGTACTCGA

sh#2	AGTACAGCCGCTCAAATCAATGCTTTTTG	GTACAGCCGCTCAAATCAATGC
HDAC3 sh#1	CCGGAAGATGCTGAACCATGCACCTCTCG AGAGGTGCATGGTTCAGCATCTTTTTTT	AATTAAAAAAAGATGCTGAACCATGCACCTCTCGA GAGGTGCATGGTTCAGCATCTT
HDAC3 sh#2	CCGGCAAGAGTCTTAATGCCTTCAACTCG AGTTGAAGGCATTAAGACTCTTGTTTTT	AATTAAAAACAAGAGTCTTAATGCCTTCAACTCGAG TTGAAGGCATTAAGACTCTTG

Table 5: qRT-PCR primer Sequences for ChIP analysis

Gene	5' Primer	3' Primer
CCND1	GCATCAGAGGATTCATACTG	CTCCTGTGTGAATTCGC
MDM2	GGAGTTAAGTCCTGACTTGTCT	GATCAGCAGAGAAAAAGTGGC
SKI	CAGAAGACGCTGGAGCAGTTCC	CTTGGCGCTCTCCTTCTTGTAG
KIF1B	TCCTCCGCAGCCGTCAC	CATCACGCAGCAGCCCG
BCL6	CCTCTTATGTCACCGAATAGC	GGTTCCGGTTCGAGGCTC
GLUT1	GGTCCTATAAACGCTACGGTCC	CGTTTGGTCTCCTGCTCCCTG
NEDD9	GAAGCACAGAGAGGAAATGA	CTAGAGGCAGCTGTAAGC
PTPRF	TCCAGCTTCGGCTCCGGC	TCCACCTGGTCCCGCTCC

Supplemental Experimental Methods

Cell Culture and Transfection

All mammalian cells used in this study were cultured in DMEM (Invitrogen, USA), supplemented with 10% FBS (Gibco, USA) and 100 U/ml Penicillin-Streptomycin (Gibco, USA). Cells were maintained in a humidified incubator at 37°C and 5% CO_2. Sf9 cells were cultured in Grace's Insect Media (Himedia, India), supplemented with 10% FBS (Gibco, USA) and 7 µg/mL gentamicin (Gold Biotechnology, USA). For transfection in mammalian cells, Lipofectamine2000 (Invitrogen, USA) was used as per manufacturer's protocol. Sf9 transfections were performed using Cellfectin II reagent (Invitrogen, USA) as per manufacturer's protocol. For all assays, unless otherwise mentioned, cells were harvested 48 hours post transfection.

Creation of different plasmid constructs

The cDNAs for ELL and DBC1 were purchased commercially (Open Biosystems, USA). The genes were cloned into different epitope tagged pcDNA5/FRT/TO plasmids for mammalian expression and generation of stable cell lines. Deletion constructs of ELL as well as DBC1 were also created in epitope tagged pcDNA5/FRT/TO vectors. For baculovirus-mediated expression in insect cells, genes were cloned in pFASTBAC vector as mentioned. For His-tagged and GST tagged protein expression in bacteria, genes were cloned in 6xHis pET-11d and pGEX vectors respectively. Site directed mutagenesis was performed using the QuikChange II Site-Directed Mutagenesis Kit (Agilent technologies, USA). Details of all clones are available upon request from the corresponding author.

Stable Cell line Generation

Generation of stable mammalian cell line for FLAG-HA-ELL has been described previously (1). For creation of stable lines expressing FLAG-HA-DBC1, essentially the same protocol was followed. Briefly, after transfection of Flp-In T-REx 293 cells with the respective plasmid, along with pOG44, cells were subjected to 200 µg/ml Hygromycin (Invitrogen, USA) selection for several weeks until individual colonies were obtained. Individual colonies were further amplified and then screened for the expression of the protein of interest. Cells were harvested in 1X Phosphate buffered saline (PBS, 137mMNaCl, 2.7mMKCl, 8mM Na_2HPO_4, and 2mM KH_2PO_4) and lysed in RIPA Buffer (10mMTris-Cl (pH 8.0), 1mM EDTA, 1% Triton X-100, 0.1% SDS, 150mMNaCl). Expression of target proteins was checked via SDS-PAGE followed by probing with epitope tag-specific antibodies.

Mass Spectrometry Analysis

Mass spectrometry analyses of DBC1 associated proteins was performed as described previously (1). Purified DBC1.com was resolved on a 4-12% SDS-PAGE gel and proteins were stained using Coomassie Brilliant Blue-G. Protein bands were excised carefully and destained in 20% methanol for 6 hours. Cysteines were reduced and alkylated with IAA. Proteins were then digested overnight with modified Trypsin (sequencing grade). The peptides were resolved on a nanocapillary reverse phase column using a 1% acetic acid/acetonitrile gradient at 400nl/min rate flow. The separated peptides were injected into a linear ion-trap mass spectrometer (LTQ XL, ThermoFisher). MS/MS spectra of the most were collected and proteins identified by comparing the data against Human IPI database (v 3.41) using X!Tandem/Trans-Proteomic Pipeline (TPP) software suite.

Nuclear Extract Preparation

Nuclear extract preparation from cells was done as described earlier (1). Briefly, cells were harvested in PBS and centrifuged at 800x*g* for 5 minutes. The packed cell volume (PCV) was estimated and cells were resuspended in 2xPCV of Buffer NE1 (10mM Tris-Cl pH 7.3, 1.5mM $MgCl_2$, 10mMNaCl, 0.7 µl/ml β-Mercaptoethanol. After leaving on ice for 15 minutes, the cells were drawn into a syringe with a 23 gauge needle, and repeatedly injected and aspirated 8 times in order to shear the cell membrane. The lysate was centrifuged at 6000 rpm for 5 minutes at 4^oC to obtain the nuclear pellet. After estimation of the nuclear pellet volume (NPV), the pellet was resuspended in 0.5xNPV of Buffer NE2 (20mM Tris-Cl pH 7.3, 1.5mM $MgCl_2$, 20mM NaCl, 0.2mM EDTA, 25% Glycerol, 0.7 µl/ml β-Mercaptoethanol and Protease inhibitor Cocktails (PIC) (Roche, USA). An equal volume of NE3 Buffer (20mM Tris-Cl pH 7.3, 1.5mM $MgCl_2$, 1.2M NaCl, 0.2mM EDTA, 25% Glycerol, 0.7 µl/ml β-Mercaptoethanol and PIC) was then added and the cells were vortexed immediately to ensure uniform mixing. The resulting homogenate was incubated on ice for 45 minutes and vortexed every 3 minutes for mixing and subsequent extraction from nuclei. Finally, the nuclear lysate was centrifuged at 12000x*g* for 20 minutes at 4^oC to obtain the supernatant containing the nuclear extract. The extract was either immediately utilized for experimental procedures or flash frozen and stored at -80^oC for future use.

Immunoprecipitation analyses

For exogenously expressed epitope tagged proteins, cells were harvested 48 hours post transfection and lysed in BC300 buffer (20mM Tris-Cl, pH 8.0, 300mM KCl, 2mM EDTA, 20% Glycerol). The cell

supernatant containing the total protein was subjected to immunoprecipitation using anti-epitope antibody-tagged magnetic/agarose beads. After incubation, the beads were washed thrice with BC300 + 0.1% NP40 to remove unbound proteins and non specific interactors. The beads were then boiled in 1X SDS loading dye at 95°C for 10 minutes to elute bead bound proteins, which were subsequently analyzed by Western blotting using tag/protein-specific antibodies.

For endogenous immunoprecipitation experiments, nuclear extract was prepared from 293T cells as described previously. The extract, containing approximately 500µg of proteins was subjected to pre-clearing using Protein-A agarose beads for 2hours at 4°C. The beads were then discarded and the pre-cleared cell lysate was incubated with 2µg of the indicated primary antibody overnight at 4°C. The antibody bound proteins were then immunoprecipitated using Protein G magnetic beads (BioRad, USA) at 4°C for 1 hour. The supernatant was discarded and the beads were washed 3X using the buffer used for nuclear extract preparation. Subsequently, the beads were boiled in 1X SDS loading dye to at 95°C for 10 minutes to elute bound proteins, which were analyzed by SDS-PAGE followed by Western blotting.

DBC1.com complex purification from nuclear extract

Flag-HA tagged DBC1 expressing stable cells were grown in a large volume (~ 6 litres) in a spinner flask. Nuclear extract was prepared as per the method described earlier. The extract was pre-cleared using Protein-Aagarose beads for 2 hours at 4°C. The pre-cleared extract was incubated with Anti-FLAG M2 Magnetic Beads (Sigma, USA) overnight at 4°C. Bead bound proteins were subjected to extensive washing with BC300 buffer. Finally, bound proteins were eluted by incubating with 3X FLAG peptide (250ng/µl) for 1 hour at 4°C. The purified complexes were separated on a 4-12% gel and stained using ProteoSilver Silver Stain Kit(Sigma, USA).

Luciferase Reporter Gene Assay

Luciferase assays were carried out using the G-293T cell line (2). G-293T is a HEK-293T-based cell line containing a chromosomally integrated luciferase gene downstream of Gal4 binding sites. For the assay, cells were transfected with the mentioned amount of the respective constructs along with Gal4-VP16 activator. 48 hours post transfection, cells were harvested as per the protocol mentioned in Dual-Glo® Luciferase Assay System (Promega, USA) and luciferase activity measured using the GloMax 20/20 Luminometer (Promega, USA).

Cycloheximide Chase Assay

Cells were transfected with the indicated constructs in the combinations mentioned. 24 hours post transfection, cells were treated to 100 µg/ml cycloheximide (Sigma, USA). At the indicated time points, cells were harvested in ice-cold PBS and lysed using RIPA buffer. Lysates were analysed by western blotting with respective antibodies.

Recombinant protein purification

For the purpose of *in vitro* reactions and interaction analyses, proteins were purified either from bacterial or mammalian cells. For purification of proteins from bacteria, *E. coli* BL21-DE3 cells expressing the protein of interest were grown for 4 hours after induction with 1mM IPTG (Goldbio, USA). Harvested cells were lysed in appropriate buffer by sonication [60% amplitude with 10 cycles of alternating on/off phases]. The cell supernatant containing the protein of interest was incubated with Ni-NTA beads for His-tagged proteins and GST agarose beads for GST-tagged proteins. The beads were then washed with appropriate buffers, and eluted using GST elution buffer or His elution buffer.

In vitro acetylation assay

Purified ELL was incubated with purified p300 HAT domain and 0.5mM acetyl-CoA in HAT buffer (75mMTris-Cl (pH 8.0), 1.25mM EDTA, 12.5mM DTT, 0.25% Tween20, 25% glycerol). The reaction mix was incubated at 30°C for 2 hours with intermittent tapping. The reaction was stopped by adding SDS loading dye, followed by boiling at 95°C for 10 minutes. The products were analysed by SDS PAGE followed by immunoblotting using pan acetyl lysine-specific antibody.

***In vitro* deacetylation assay**

Flag tagged HDAC3 was purified by immunoprecipitation from 293T cells. Purified acetylated ELL was incubated with HDAC3 in HDAC buffer (50mMTris [pH 8.0], 4mM $MgCl_2$, 0.2mM DTT) on a rotor for 3 hours at 37°C. The reaction was stopped by adding SDS loading dye and boiling the samples for 10 minutes at 95°C. Proteins were resolved by SDS-PAGE and analyzed by western blotting.

NGS data mining and GO analysis

Identification of target genes for both DBC1 and ELL was done using the publicly available datasets GSE35480 (3) and GSE34104 (4). Genes were sorted in MS Excel and overlapping genes identified using Venny. Initial gene ontology of overlapping genes was performed using Protein Annotation Through Evolutionary Relationship (PANTHER) software (5). Further functional classification and disease annotation of common genes was done using Database for Annotation, Visualization and Integrated Discovery (DAVID) (6).

Generation of stable knockdown cells

For the purpose of generating cells with DBC1 stably knocked down, DBC1 specific shRNAs were cloned into pLKO.1 vector. 375ng psPAX2 packaging plasmid, 125ng pMD2.G VSV-G envelope plasmid and 500ng of the respective shRNAs were co-transfected into $5x10^5$cells in a 6 well plate. 72 hours post transfection, the supernatant containing virus particles was collected, centrifuged briefly and used to transduce fresh cells. Polybrene was added at a concentration of 8 µg/ml to aid viral transduction. Cells were then subjected to selection with 3 µg/ml puromycin. Puromycin resistant cells were amplified and screened to identify knock down cell lines via immunoblotting and qRT-PCR analysis.

qRT PCR for gene expression analysis

For qRT-PCR assay, total RNA was extracted from cells using TRIzol as per manufacturers protocol. 1 µg of total RNA was used as a template for cDNA synthesis using Verso cDNA synthesis kit (Thermo Fisher Scientific, USA). cDNA was diluted 25 times using Ultrapure water (Life Technologies, USA). qRT-PCR was performed in a CFX96 Touch Real-Time PCR Detection System (BioRad, USA) usingiTaq Universal SYBR Green Supermix (BioRad, USA) and specific primers for target genes. Relative expression was calculated by normalizing the gene expression to 18s rRNA expression as an internal control.

Chromatin Immunoprecipitation Assay

Chromatin immunoprecipitation (ChIP) assays were performed as described earlier (2). Cells were cross-linked using 1% formaldehyde (Sigma, USA) for 10 minutes at room temperature. After a brief wash with cold 1X PBS, quenching was done by addition of 125mM glycine. After another brief wash with cold PBS, cells were harvested with a scraper and centrifuged at 2500 RPM for 10 minutes. Nuclear fraction was prepared by lysing the cells in nuclear extraction buffer(0.5% NP40, 1% Triton X-100, 300mM NaCl, 20mM Tris (pH 7.5), 2mM EDTA and protease inhibitor cocktail (PIC)). The nuclear fraction thus obtained was resuspended in shearing buffer (1% SDS, 50mM Tris (pH 8.1), 10mM EDTA and PIC). Sonication was performed in a UCD-200 Bioruptor (Diagenode) for 30 min with alternating on/off pulses at 30 seconds interval. The sonicated chromatin was checked on a 1% agarose gel to ensure DNA fragmentation between 200-500 bp. Following sonication, the samples were pre-cleared by incubation with Protein Aagarose beads at 4°C. The pre-cleared lysate was diluted 10 times with ChIP dilution buffer (0.01% SDS, 1.1% Triton X-100, 1.1mM EDTA, 20mM Tris-Cl (pH 8.0) and 167mM NaCl). Immunoprecipitations were performed overnight at 4°C using 2 µg of the respective primary antibodies. The antibody bound complexes were then incubated with Protein G magnetic beads (BioRad, USA) at 4°C for 1 hour. The bead bound complexes were then washed with low salt buffer (0.1% SDS, 1% TritonX-100, 2mM EDTA, 20mM Tris-Cl (pH 8.0), 150mM NaCl, and PIC), followed by high salt buffer (0.1% SDS, 1% Troton X-100, 2mM EDTA, 20mM Tris-Cl (pH 8.0), 500mM NaCl, and PIC) and LiCl buffer (0.5M LiCl, 1% NP40, 1% deoxycholate, 20mMTrisCl(pH8.0) and 1mM EDTA). DNA complexes were eluted by incubation with elution buffer (1% SDS, 0.1M $NaHCO_3$) and cross links reversed by incubation at 65°C for 4 hours. The DNA was then purified by QIAquick PCR purification kit (Qiagen, Germany) as per the recommended protocol. The purified DNA was used as the template for subsequent qRT-PCR reactions.

PBMC Isolation

For the purpose of downstream experiments, peripheral blood mononuclear cells (PBMCs) were isolated from whole blood using HiSep LSM1077 (Himedia Laboratories, India) as per the manufacturer's protocol. The HiSep Solution was carefully pipetted into sterile 15ml centrifuge tubes. Whole blood collected in EDTA coated vials was gently layered onto the HiSep solution. The ratio of HiSep: blood was maintained at 1:1. The tubes were centrifuged at 500*g*for 30 minutes at 25 °C. The interface of plasma and HiSep, containing the PBMCs was aspirated carefully and washed thrice with 1x PBS. They were then lysed using RIPA buffer for obtaining proteins, or used for RNA extraction with TRIzol.

References:

1. Biswas D*, et al.* (2011) Function of leukemogenic mixed lineage leukemia 1 (MLL) fusion proteins through distinct partner protein complexes. *Proc Natl Acad Sci U S A* 108(38):15751-15756.
2. Ghosh K*, et al.* (2018) Positive Regulation of Transcription by Human ZMYND8 through Its Association with P-TEFb Complex. *Cell Rep* 24(8):2141-2154 e2146.
3. Close P*, et al.* (2012) DBIRD complex integrates alternative mRNA splicing with RNA polymerase II transcript elongation. *Nature* 484(7394):386-389.
4. Byun JS*, et al.* (2012) ELL facilitates RNA polymerase II pause site entry and release. *Nat Commun* 3:633.
5. Mi H, Guo N, Kejariwal A, & Thomas PD (2007) PANTHER version 6: protein sequence and function evolution data with expanded representation of biological pathways. *Nucleic Acids Res* 35(Database issue):D247-252.
6. Huang da W, Sherman BT, & Lempicki RA (2009) Systematic and integrative analysis of large gene lists using DAVID bioinformatics resources. *Nat Protoc* 4(1):44-57.
